# An interaction between synapsin and C9orf72 regulates excitatory synapses and is impaired in ALS/FTD

**DOI:** 10.1007/s00401-022-02470-z

**Published:** 2022-07-25

**Authors:** Claudia S. Bauer, Rebecca N. Cohen, Francesca Sironi, Matthew R. Livesey, Thomas H. Gillingwater, J. Robin Highley, Daniel J. Fillingham, Ian Coldicott, Emma F. Smith, Yolanda B. Gibson, Christopher P. Webster, Andrew J. Grierson, Caterina Bendotti, Kurt J. De Vos

**Affiliations:** 1 Sheffield Institute for Translational Neuroscience (SITraN), Department of Neuroscience, University of Sheffield, 385a Glossop Road, Sheffield, S10 2HQ UK; 2 Neuroscience Institute, University of Sheffield, Western Bank, Sheffield, S10 2TN UK; 3 Laboratory of Molecular Neurobiology, Department of Neuroscience, Istituto di Ricerche Farmacologiche Mario Negri IRCCS, Via Mario Negri 2, 20156 Milan, Italy; 4 Edinburgh Medical School: Biomedical Sciences, University of Edinburgh, Hugh Robson Building, Edinburgh, EH8 9XD UK; 5 Euan MacDonald Centre for Motor Neuron Disease Research, Chancellor’s Building, University of Edinburgh, Edinburgh, EH16 4SB UK

**Keywords:** C9orf72, Amyotrophic lateral sclerosis, Frontotemporal dementia, Synapsin, Synapse

## Abstract

Dysfunction and degeneration of synapses is a common feature of amyotrophic lateral sclerosis and frontotemporal dementia (ALS/FTD). A GGGGCC hexanucleotide repeat expansion in the *C9ORF72* gene is the main genetic cause of ALS/FTD (C9ALS/FTD). The repeat expansion leads to reduced expression of the C9orf72 protein. How C9orf72 haploinsufficiency contributes to disease has not been resolved. Here we identify the synapsin family of synaptic vesicle proteins, the most abundant group of synaptic phosphoproteins, as novel interactors of C9orf72 at synapses and show that C9orf72 plays a cell-autonomous role in the regulation of excitatory synapses. We mapped the interaction of C9orf72 and synapsin to the N-terminal longin domain of C9orf72 and the conserved C domain of synapsin, and show interaction of the endogenous proteins in synapses. Functionally, C9orf72 deficiency reduced the number of excitatory synapses and decreased synapsin levels at remaining synapses in vitro in hippocampal neuron cultures and in vivo in the hippocampal mossy fibre system of C9orf72 knockout mice. Consistent with synaptic dysfunction, electrophysiological recordings identified impaired excitatory neurotransmission and network function in hippocampal neuron cultures with reduced C9orf72 expression, which correlated with a severe depletion of synaptic vesicles from excitatory synapses in the hippocampus of C9orf72 knockout mice. Finally, neuropathological analysis of post-mortem sections of C9ALS/FTD patient hippocampus with C9orf72 haploinsufficiency revealed a marked reduction in synapsin, indicating that disruption of the interaction between C9orf72 and synapsin may contribute to ALS/FTD pathobiology. Thus, our data show that C9orf72 plays a cell-autonomous role in the regulation of neurotransmission at excitatory synapses by interaction with synapsin and modulation of synaptic vesicle pools, and identify a novel role for C9orf72 haploinsufficiency in synaptic dysfunction in C9ALS/FTD.

**Supplementary Information:**

The online version contains supplementary material available at 10.1007/s00401-022-02470-z.

## Introduction

Amyotrophic lateral sclerosis and frontotemporal dementia (ALS/FTD) are two closely related late-onset neurodegenerative disorders that overlap genetically, pathologically and clinically. ALS is characterised by selective demise of upper and lower motor neurons leading to progressive paralysis, whereas FTD is typified by degeneration of prefrontal cortical neurons that commonly gives rise to cognitive and behavioural impairments. In addition, extra-motor and other prefrontal regions such as the hippocampus are now being implicated in the pathogenesis of ALS/FTD [60, 82]. A GGGGCC (G4C2) repeat expansion in intron 1 of the *C9orf72* gene is the most common genetic cause of ALS/FTD (C9ALS/FTD) [17, 61]. The exact mechanisms by which the repeat expansion causes C9ALS/FTD are unknown, but there is evidence for both loss- and gain-of-function. Three non-exclusive mechanisms have been proposed, namely loss of C9orf72 function due to the G4C2 repeat expansion causing decreased C9orf72 expression (C9orf72 haploinsufficiency), RNA toxicity caused by sequestration of RNA-binding proteins to sense and anti-sense repeat RNA foci, and protein toxicity deriving from non-canonical repeat associated non-AUG (RAN) translation of sense and anti-sense repeat transcripts into dipeptide repeat (DPR) proteins (Reviewed in [5]).

Recent evidence suggests a dual-hit model in which C9orf72 haploinsufficiency synergises with repeat-dependent gain-of-function mechanisms. In vitro, loss of C9orf72 increased levels of DPR proteins and thereby exacerbated protein toxicity [8]*. *In vivo, inactivation of one or both endogenous C9orf72 alleles in mice expressing human transgenes carrying the repeat expansion caused elevated DPR accumulation which was accompanied by exacerbated cognitive deficits, glial activation and hippocampal neuron loss [87] and C9orf72 deficiency promoted motor deficits of a C9ALS/FTD BAC transgenic mouse model in a gene dosage-dependent manner [65]. Understanding the pathophysiological impact of C9orf72 haploinsufficiency is key to our understanding of disease progression in C9ALS/FTD.

Despite its role in disease progression, the physiological function of the C9orf72 protein still remains poorly understood. We and others have identified C9orf72 as a regulator of membrane trafficking in the autophagy pathway through interactions with Rab GTPases, the ULK1 autophagy initiation complex, and lysosomes [2, 3, 20, 34, 64, 74, 80, 81]. In addition, C9orf72 has been shown to regulate Arf family GTPases which may explain its reported role in actin dynamics and endosomal sorting [15, 20, 66, 67, 72]. Recent work showing expression of the C9orf72 protein in synapses suggests C9orf72 may serve a physiological function in synapses [21, 84]. Nevertheless, exactly how C9orf72 haploinsufficiency impacts on neuronal function and its potential contribution to ALS/FTD pathology remains unknown.

Synaptic dysfunction and degeneration are inherent pathophysiological hallmarks of neurodegenerative diseases, including ALS/FTD [30, 56, 57]. Here we have identified the synapsin protein family as novel interactors of C9orf72. Synapsins are synaptic vesicle proteins found in all presynaptic terminals which modulate neurotransmission by regulating synaptic vesicle pools [70]. We demonstrate that C9orf72 haploinsufficiency impairs excitatory synapses in vitro and in vivo as well as in post-mortem brain of C9ALS/FTD patients. Loss of C9orf72 caused depletion of synapsin from synapses and severely reduced the number of synaptic vesicles in excitatory synapses which was accompanied by impaired synaptic function and network excitability. These results reveal a novel role of C9orf72 in the regulation of synaptic vesicles and neurotransmission. Our data propose that C9orf72 haploinsufficiency is a major contributor to synaptic dysfunction in C9ALS/FTD.

## Materials and methods

### Plasmids

A modified pCI-neo vector containing a V5-tag was generated by ligating Nhe1-V5-Xho1 oligos (Sigma-Aldrich/Merck Life Science UK Ltd, Gillingham, UK) into pCI-neo (Promega UK Ltd, Southampton, UK). Syn3a (Origene, NM_003490) was amplified by PCR using Phusion High Fidelity enzyme (NEB, Hitchin, UK) and CTCGAGgccaccatgaatttcctccggcgacgt and GCGGCCGCctagtcagagaacaggctggcaaaagact forward and reverse primers and cloned into pCR-blunt-II-TOPO (ThermoFisher Scientific, Runcorn, UK) before being subcloned into the pCI-neo-V5 vector using Xho1 and Not1 restriction sites.

EGFP-Syn1a, YFP-Syn2a, and EGFP-Syn3a were a gift from Dr. Daniel Gitler, Ben-Gurion University, Israel. To generate V5-tagged Syn1a and Syn2a, Syn1a and Syn2a were PCR amplified from EGFP-Syn1a and YFP-Syn2a using CTCGAGatgaactacctgcggcgccgcct and GCGGCCGCtcagtcggagaagaggctggcgaa or CTCGAGatgatgaacttcctgcggc and GCGGCCGCctaatctgaaaagaggctggc forward and reverse primers, respectively, and subcloned into pCI-neo-V5 via pCR-blunt-II-TOPO.

EGFP-Syn3a deletion mutants were generated by inserting stop codons at the end of domain A (E29*), B (R92*), C (S401*), and J (S530*) by site-directed mutagenesis (Genscript Biotech (UK) Ltd, Oxford, UK).

Myc-tagged C9orf72L and C9orf72S in pRK5 have been described previously [80]. To generate Myc-C9orf72ΔLGN (AA 205–481) the C-terminal DENN domain was amplified using CTCGAGgtactcaatgatgatgatatt and GCGGCCGCttaaaaagtcattagaacatc forward and reverse primers and subcloned into the pCI-neo-Myc vector using Xho1 and Not1 restriction sites via pCR-blunt-II-TOPO before being transferred to pRK5.

pCMV6entry-SMCR8-mycDDK was purchased from Origene, Rockville, MD, USA.

pCI-neo and pEGFPC2 (Clontech, Takara Bio Europe S.A.S., Saint-Germain en Laye, France) were used as empty vector (EV) or EGFP control respectively.

### Antibodies

Details of all primary antibodies used in this study are listed in Table 1. Secondary antibodies used for immunoblotting were either horseradish peroxidase (HRP)-coupled goat anti-rabbit and goat anti-mouse IgG (Dako, Agilent Technologies LDA, London, UK; 1:5000) or near-infrared fluorescent conjugated Alexa Fluor 680 donkey anti-mouse IgG and Alexa Fluor 790 donkey anti-rabbit IgG (Jackson ImmunoResearch, Stratech Scientific Ltd, Ely, UK; 1:50,000).

**Table 1:** Primary antibodies used in this study

Antibody	Host	Vendor information	Application	Comments
Actin	Mouse	MAB 1501, Merck Life Science UK Ltd, Gillingham, UK	WB: 1:5000	
C9orf72	Mouse	GTX632041, GeneTex, Insight Biotechnology Ltd, London, UK	IP: 4 µg IF and PLA: 1:500 IHC human: 1:1600	KO verified (Supplier) [42] Used to detect endogenous C9orf72 protein in Fig. 1d, e (IP); Fig. 1f, Supplementary Fig. 4, Online Resource (IF, PLA); Fig. 6 (IHC human)
C9orf72	Rabbit	25757-1-AB, Proteintech Europe Ltd, Manchester, UK	WB: 1:1000	KO verified (Supplier and Fig. 1e, Supplementary Fig. 1c, Supplementary Fig. 2a, Online Resource) Used to detect endogenous C9orf72 protein in Fig. 1d, e, Supplementary Fig. 1a, Supplementary Fig. 2a, Supplementary Fig. 4c, Supplementary Fig. 6b, Supplementary Fig. 9b Online Resource (WB)
C9orf72	Rabbit	sc-138763, Santa Cruz Biotechnology Inc., Heidelberg, Germany	WB: 1:500	Used to detect overexpressed Myc-tagged C9orf72 in Fig. 1a, b
FLAG	Mouse	Clone M2, F3165, Sigma-Aldrich/Merck Life Science UK Ltd	IP: 4 µg	
GAPDH	Rabbit	14C10, Cell Signalling Technology Europe BV, Leiden, Netherlands	WB: 1:2000	
Gephyrin	Mouse	147011, Synaptic Systems GmbH, Goettingen, Germany	IF: 1:500	KO verified (Supplier)
GFP	Mouse	Clone JL-8, 632381, Clontech, Takara Bio Europe S.A.S	WB: 1:10,000 IP: 1 µg	
GFP	Rabbit	ab6556, Abcam Plc, Cambridge, UK	WB: 1:2500	
Homer I	Rabbit	160002, Synaptic Systems	IF: 1:500	
Iba1	Goat	ab5076, Abcam	IF: 1:750	
MAP2	Chicken	NB300-213, Novus Biologicals, Bio-Techne Ltd, Oxon, UK	IF: 1:1000	
Myc	Mouse	clone 9B11, 2276S, Cell Signalling	WB: 1:2000 IF: 1:2000 IP: 0.318 µg	
Myc	Rabbit	ab9106, Abcam,	WB: 1:2000	
PDI	Mouse	GTX25484, GeneTex	WB: 1:2000	
PSD95	Mouse	Clone 6G6-1C9, MAB 1596, Merck Life Science UK Ltd	IF: 1:1000	Validated by relative expression in mouse brain v mouse liver and skeletal muscle (Supplier)
PSD95	Goat	ab12093, Abcam	IHC mouse 1:100	
SMCR8	Rabbit	GTX635798, GeneTex	WB: 1:1000	
SSRP1	Mouse	ab26212, Abcam	WB: 1:500	
Synaptophysin 1	Chicken	101006, synaptic systems	IF: 1:500	
Synapsin 1	Mouse	106001 and 106011, synaptic systems	WB: 1:1000 IF: 1:500 IHC mouse: 1:200 IHC human 1:3200	KO verified (Supplier) Used in Fig. 3 (IF), Figs. 4, 5, Supplementary Fig. 8b, Online Resource (IHC mouse), Fig. 6 (IHC human), Supplementary Fig. 6c, Supplementary Fig. 8a, Online Resource (WB)
Synapsin 1	Rabbit	S193, Sigma-Aldrich/Merck	IF and PLA: 1:2000	Used in Fig. 1f, Supplementary Fig. 4, 5, Online Resource (IF and PLA)
Synapsin 3	Rabbit	106303, Synaptic Systems	WB: 1:1000 IF and PLA: 1:500 IHC mouse 1:500	KO verified (Supplier)
SV2	Mouse	SV2, Developmental Studies Hybridoma Bank (DSHB), University of Iowa, IA, USA	IF: 1:1000 IHC: 1:200 WB: 1:1000	KO verified [16]
VGAT	Rabbit	131003, Synaptic Systems	IF: 1:500	KO verified (Supplier)
Vimentin	Chicken	AB5733, Merck Life Science UK Ltd	IF: 1:1000	
V5	Rabbit	Clone D3H8Q, 13202, Cell Signalling	WB: 1:500	
V5	Mouse	MA5-15253, Thermo Fisher Scientific, Runcorn, UK	WB: 1:1000 IF: 1:1000	

Secondary antibodies used for immunofluorescence were Alexa fluorophore (488, 568, or 633)-coupled goat or donkey anti-mouse IgG, Alexa fluorophore (488, 568, or 633)-coupled goat or donkey anti-rabbit IgG, Alexa fluorophore (488, 568, 647)-coupled donkey anti-goat IgG (All from Invitrogen, ThermoFisher Scientific; 1:500), or DyLight 405-coupled goat anti-chicken or Cy5-coupled donkey anti-chicken (both from Jackson ImmunoResearch, 1:500).

### Yeast-2-hybrid

Human C9orf72L was cloned as a “bait” fused to the DNA binding domain of GAL4 in pGBKT7 using EcoRI/BamHI restriction sites. This construct was used to screen an arrayed human brain full-length open reading frame (ORF) cDNA library containing approximately 15,000 ORF clones by Protein Interaction Screening, Genomics and Proteomics Core Facilities (W150), German Cancer Research Center (DKFZ), Heidelberg, Germany [39].

### Cell culture

HEK293 were cultured in Dulbecco's modified Eagle's medium (DMEM, Sigma-Aldrich/Merck Life Science UK Ltd) supplemented with 10% FBS (Labtech International Ltd, Heathfield, UK) and 1 mM sodium pyruvate (Sigma-Aldrich/Merck Life Science UK Ltd) in a 5% CO_2_ atmosphere at 37 °C. Cells were transfected with plasmid DNA using Lipofectamine 2000 (Invitrogen) or polyethylenimine (PEI) (stock 1 mM; 3 μl/μg plasmid).

Hippocampal neurons from E17 Sprague Dawley rat embryos (Charles River UK Ltd, Margate, UK) were isolated and cultured at a density of 50,000 cells/cm^2^ on glass coverslips or tissue culture plasticware coated with poly-d-lysine (P6407, Sigma-Aldrich/Merck Life Science UK Ltd) in Neurobasal™ medium supplemented with B27 supplement (Invitrogen, ThermoFisher Scientific), 100 IU/ml penicillin, 100 mg/ml streptomycin, and 2 mM l-glutamine as described previously for cortical neurons [80]. Half of the medium was replaced by fresh medium every 3–4 days from 5 days in vitro (5DIV) onwards.

To adequately support excitatory and inhibitory neuronal network activity in vitro [6], neurons used for electrophysiological and multi-electrode array recordings were grown from 5DIV onwards in BrainPhys™ medium with NeuroCult™ SM1 Neuronal supplement (STEMCELL Technologies UK Ltd, Cambridge, UK) and 100 IU/ml penicillin, 100 mg/ml streptomycin.

### miRNA and lentiviral production

miRNA targeting rat C9orf72 was designed and cloned as described previously [80]. Non- targeting miRNA was part of the BLOCK-i™ Pol II miR RNAi expression vector kit. Lentivirus was made in-house as described previously [80] or produced by VectorBuilder Inc. (Chicago, IL, USA) using the same miRNA vectors. For lentiviral transduction, neurons at 5DIV were exposed to 4 TU/cell of in-house produced lentivirus or to 30 TU/cell of commercially produced virus in completely fresh culture medium overnight. The next morning, transduction medium was replaced by 50% conditioned/50% fresh culture medium.

### RNA extraction and RT-qPCR for lentiviral knockdown efficiency

To validate lentiviral knockdown efficiency, RNA was extracted from hippocampal neurons on 12DIV and transcribed into cDNA as described previously [80]. RT-qPCR reactions were performed in a Bio-Rad CFX96 Touch thermocycler (Bio-Rad Laboratories Ltd., Watford, UK). Samples were amplified in triplicate using HOT FIREpol EvaGreen qPCR Mix Plus (Solis BioDyne OU, Tartu, Estonia) and 500 nM of each optimized forward and reverse primer. Cycling conditions were as follows: 95 °C for 5 min to denature followed by 39 cycles of 95 °C for 30 s and 60 °C for 1 min. RT-qPCR cycles were followed by a melt curve cycle to ensure single products were generated. Conditions for the melt curve were 1 min at 95 °C, followed by 60 cycles of 0.5 °C/cycle from 65 °C. Data were analysed in Bio-Rad CFX Manager software and mRNA levels determined comparatively to control via the ∆∆Ct method [47]. Primer sequences were as follows: rat RPL19 FW: ctcgatgccggaagaacacc, REV: gagcgttggcagtaccctt; and rat C9orf72 FW: gtgttgacaggctaacgcac, REV: agggatgacc tccccagtaa.

### Protein extraction

To validate knockdown of C9orf72 protein by immunoblot, hippocampal neurons at 12DIV were washed in phosphate buffered saline (PBS, 137 mM NaCl, 2.7 mM KCl, 1.5 mM KH_2_PO_4_, 10 mM Na_2_PO_4_.2H_2_O), scraped into Laemmli buffer (60 mM Tris HCl pH 6.8, 2% SDS, 0.002% bromophenol blue, 5% β-mercaptoethanol, 5% glycerol) and passed through a 25 G needle. Protein samples were denatured at 100 °C for 5 min before analysis by immunoblot.

### Immunoprecipitation

HEK293 cells were washed in PBS, harvested into modified BRB80 buffer (80 mM PIPES pH 6.8, 150 mM NaCl, 1 mM MgCl_2_, 1 mM EDTA, 1% w/v NP-40, 1 × Halt™ Protease Inhibitor Cocktail (ThermoFisher Scientific)) and lysed on a roller at 4 °C for 1 h. Lysates were cleared by centrifugation at 15,000×*g* for 30 min at 4 °C, protein concentration measured by Bradford protein assay (Bio-Rad) and 0.5–1 mg protein was incubated with primary antibody overnight at 4 °C. 20 µl Protein G Sepharose beads (GE Healthcare) were added to the samples and incubated for 2 h at 4 °C. Alternatively, EGFP- or DDK (FLAG)-tagged proteins were immunoprecipitated using 10 μl GFP-Trap magnetic agarose beads (Chromotek GmbH, Planegg, Germany) or FLAG M2 magnetic agarose beads (Sigma-Aldrich/Merck). For this 1 mg of protein was incubated at 4 °C for 1 h or overnight. Beads were washed with buffer and eluted into Laemmli. Magnetic beads were harvested using an Extractman device (Gilson Scientific Ltd, Dunstable, UK).

For endogenous immunoprecipitation of C9orf72, synaptosomes were prepared by differential centrifugation as described previously [37]. Briefly, mouse brains were homogenised in 0.5 ml of sucrose buffer (0.32 M sucrose, 3 mM HEPES-Na, pH 7.4, 0.1 mg/ml phenylmethylsulfonyl fluoride (PMSF), 250 µM DTT, Halt™ Protease Inhibitor Cocktail, 1 × PhosSTOP™ (Merck Life Science)). The homogenate was centrifuged at 1000×*g* at 4 °C for 10 min to produce pellet (P1) and supernatant (S1). P1 pellet was resuspended in 0.5 ml sucrose buffer and centrifuged at 1000×*g* at 4 °C for 10 min to obtain a crude nuclear fraction (P1′) and supernatant (S1′). S1 and S1′ were combined and centrifuged at 12,000×*g* at 4 °C for 15 min to produce a pellet (P2) and supernatant (S2). P2 was resuspended in 0.5 ml of sucrose buffer and centrifuged for 15 min at 13,000×*g* at 4 °C to yield a crude synaptosomal fraction (P2’). S2 was centrifuged at 33,000×*g* at 4 °C for 20 min to obtain the microsomal pellet P3. For endogenous IP, pellet P2′ was solubilised in endogenous IP buffer (80 mM PIPES pH 6.8, 1 mM MgCl_2_, 1 mM EDTA, 1% (w/v) NP-40, 1 × Halt™ Protease Inhibitor Cocktail, 1 × PhosSTOP™) for 60 min. Lysates were cleared at 12,000×*g* for 30 min at 4 °C and 1 mg of protein was incubated with 4 µg of primary antibody in IP buffer supplemented with 50 mM NaCl for 16 h at 4 °C. Antibody was then captured by incubation with 15 µl Protein G Mag Sepharose™ Xtra (Cytiva, Fisher Scientific UK Ltd, Loughborough, UK) beads for 2.5 h at 4 °C. Samples were denatured at 100 °C and loaded onto polyacrylamide gels.

### SDS-PAGE and immunoblotting

Proteins were separated by SDS–PAGE and transferred to nitrocellulose membranes (Whatman, Fisher Scientific) by electroblotting (Bio-Rad). After transfer, membranes were blocked for 1 h at room temperature in Tris-buffered saline (TBS) with 5% fat-free milk (Marvel, Sainsbury’s, London, UK) and 0.1% Tween-20. Membranes were incubated with primary antibodies in blocking buffer for 1 h at room temperature or overnight at 4 °C. Membranes were washed 3 times for 10 min in TBS with 0.1% Tween-20 before incubation with HRP- or near-infrared AlexaFluor-coupled secondary antibodies in TBS with 0.1% Tween-20 for 1 h at room temperature. After washing, membranes were prepared for chemiluminescent signal detection with SuperSignal West Pico Chemiluminescent substrate (ThermoScientific, Fisher Scientific) according to the manufacturer’s instructions. Signals were detected with a SynGene Gbox (Syngene International Ltd, Bangalore, India) or on ECL film (GE Healthcare, Fisher Scientific). Signal intensities were quantified using ImageJ/Fiji (http://imagej.nih.gov/ij/) [63]. Alternatively, near-infrared fluorescent signals were detected using an Odyssey XF Imaging System with ImageStudio software (LI-COR Biosciences UK Ltd, Cambridge, UK).

### Immunofluorescence and proximity ligation assays

Immunostaining was performed as described previously [80]. Briefly, HEK293 cells or hippocampal neurons at 12DIV grown on glass coverslips were fixed with 3.7% formaldehyde in PBS for 20 min at room temperature. Coverslips were washed with PBS, residual formaldehyde quenched with 50 mM NH_4_Cl in PBS and washed again. Cells were permeabilized by incubation with 0.2% Triton X-100 in PBS for 3 min, blocked with PBS containing 0.2% fish gelatin for 30–60 min at room temperature and incubated with primary antibody in blocking solution for 1 h at RT or overnight at 4 °C. Coverslips were washed, incubated with secondary antibody in blocking solution for 60 min at room temperature, washed with PBS, stained with Hoechst 33342, washed and finally mounted in fluorescence mounting medium (Dako, Agilent Technologies). For staining of endogenous C9orf72, hippocampal neurons at 12DIV were fixed with ice-cold methanol for 20 min at − 20 °C, washed with PBS and permeabilised/blocked in TBS with 5% BSA and 0.3% Triton X-100 [42] for 60 min at room temperature followed by incubation in TBS with 5% BSA, 0.3% Triton X-100 and primary antibodies over night at 4 °C. In situ proximity ligation assays on HEK293 cells and hippocampal neurons were performed with the Duolink In Situ Kit following the manufacturer’s protocol (Sigma-Aldrich/Merck). Samples were mounted in mounting medium and imaged within 36 h.

Images were recorded using appropriate filter sets (Omega Optical Inc., Brattleboro, VT, USA and Chroma Technology Corp., Rockingham, NC, USA) on a Zeiss Axioplan2 microscope (Carl Zeiss Ltd., Cambridge, UK) fitted with a Hamamatsu C4880-80 (Hamamatsu Photonics UK Ltd., Welwyn Garden City, UK) or Retiga R3 (QImaging, Cairn Research Ltd., Faversham, UK) CCD camera, PE-300 LED illumination (CoolLED Ltd., Andover, UK), and a 63×, 1.4NA Plan Apochromat objective (Zeiss) or a Zeiss Axiovert 200 microscope (Zeiss) equipped with a Hamamatsu C9100-12 EMCCD, PE-4000 LED illumination (CoolLED), and 63×, 1.4NA Plan Apochromat and 100×, 1.3NA Plan Apochromat objectives (Zeiss) using MicroManager 1.4 software [19] or on an Olympus IX73 (Olympus Lifescience, Evident Europe GmbH, Stansted, UK) equipped with a Zyla 4.2 sCMOS camera (Andor Technology, Belfast, UK), SpectraX light engine (Lumencor Inc., Beaverton, OR, USA) and OptoLED (Cairn Research) illumination, and 60×, 1.35NA Universal Plan Super Apochromat and 40×, 1.35NA Universal Apochromat objectives (Olympus) using MetaMorph software (Molecular Devices Ltd., New Milton, UK)). Confocal images of immunostained cells were taken using a Leica SP5 Confocal Microscope (Leica Microsystems (UK) Ltd., Milton Keynes, UK) with the 63 × 1.2NA objective. Illumination intensities, exposure times, and camera settings were kept constant during experiments.

### Electrophysiology and analysis

Before electrophysiological recordings were carried out, hippocampal neurons at 11–12DIV were cultured overnight in 90% SGG [comprising, in mM; 114 NaCl, 5.292 KCl, 1 MgCl_2_, 2 CaCl_2_, 10 HEPES, 1 glycine, 30 Glucose, 0.5 Na-pyruvate, 0.219% NaHCO_3_, 0.1% phenol red)/10% MEM (Invitrogen)]. Patch-clamp recordings were performed at 12–13DIV as described by [48]. Briefly, electrodes were filled with (in mM): 155 K-gluconate, 2 MgCl_2_, 10 Na-HEPES, 10 Na-PiCreatine, 2 Mg_2_-ATP, and 0.3 Na_3_-GTP, pH 7.3, 300 mOsm. For mEPSC recordings, cells were bathed in an extracellular recording solution comprising (in mM): 152 NaCl, 2.8 KCl, 10 HEPES, 2 CaCl_2_, 1.5 MgCl_2_, 10 glucose, pH 7.3, 320–330 mOsm in the presence of TTX (300 nM), strychnine (20 µM) and PTX (50 µM). Recordings made at − 70 mV (− 84 mV with liquid junction potential correction), low-pass filtered online at 2 kHz, digitized at 10 kHz and recorded to a computer using WinEDR V2 7.6 Electrophysiology Data Recorder (J. Dempster, Department of Physiology and Pharmacology, University of Strathclyde, UK). Synaptic data was analysed as described previously [57]. Briefly, data was obtained from at least 2-min recordings and neurons that displayed mEPSC frequencies under 0.05 Hz were omitted from the analysis. mEPSC recordings were analysed offline using the WinEDR software. A dead time window of 10 ms was set and individual mEPSCs were detected using an algorithm that selected for mEPSCs below a − 4 to − 6 pA amplitude threshold and greater than 1 ms in duration. mEPSCs that had a monotonic rising phase with a 10–90 rise time of lower than 6 ms and a Ƭ-decay with a decay time constant of lower than 25 ms were selected for analysis. Recordings were then visually inspected for validity.

For extracellular multi-electrode array (MEA) electrophysiology, hippocampal neurons were cultured on glass arrays with 59 channels per array (60MEA200/30iR-Ti-gr; Multi Channel Systems MCS GmbH, Reutlingen, Germany). Data were recorded from cultures bathed in 90% SGG/10% MEM medium using a MEA recording platform (Multi Channel Systems) at a sampling rate of 20 kHz. Data were filtered offline with a 2nd order Butterworth filter with a cut-off frequency of 200 Hz. Data was analysed using the Multi Channel Systems software. For each MEA, a minimum of 10 active channels per array were selected for further analysis. Spikes were identified as activity 5 times the standard deviation of the baseline. Individual burst detection parameters: maximum interspike interval (ISI) to start burst, 0.5 s; maximum ISI to end burst, 0.5 s; maximum beginning ISI, 0.1 s; minimum interburst interval (IBI), 0.8 s; minimum burst duration, 0.05 s; and minimum number of spikes in a burst, 3. Synchronised network bursts throughout the array were determined using the software that detected defined individual bursts on each array that began within 0.2 s of each other. Data were determined to be parametric or non-parametric before performing statistical analysis.

### Generation of C9orf72 knockout mice

C9orf72 KO mice were generated using the Cre-LoxP system. A floxed mouse line for the conditional deletion of C9orf72 exons 3–4, coding for the two C9orf72 ATG start codons, was generated in a mixed C57BL/6 and 129Sv strain (GenOway S.A., France). These floxed mice were cross-bred with CRE Deleter C57BL/6J mice constitutively expressing the Cre recombinase under the cytomegalovirus (CMV) promoter (Taconic Biosciences GmbH, Koeln, Germany) to generate a constitutive knockout model for the C9orf72 gene (C9orf72-KO). The resulting heterozygous mice were interbred to obtain both homozygous C9orf72-KO mice and age-matched wild type (WT) controls.

All mice were maintained on the C57BL/6J genetic background and back crossed at least 6 generations. Excision of exons 3 and 4 was confirmed by qualitative PCR (Supplementary Fig. 1a, Online Resource) on DNA isolated from tail biopsies. RT-qPCR and immunoblots of brain tissue confirmed the absence of C9orf72 mRNA and protein in the brain of C9orf72-KO mice, respectively (Supplementary Fig. 1b, c, Online Resource).

Genotyping for C9orf72 was performed through Polymerase Chain Reaction (PCR) using genomic DNA extracted from tail biopsies collected from the mice after weaning, within twenty-one days of age. Caudal biopsies were completely digested using the Maxwell® 16 System (Promega), according to the manufacturer’s instructions. The amplification solution was composed of 1X Buffer 3 (Roche), 250 µM dNTPs (Promega), 0.5 µM forward primer (5′-GCCCTCCCCTTCCTGTTTTGTTCT-3′) (Metabion International AG, Planegg/Steinkirchen, Germany), 0.5 µM reverse primer (5′-AGACGGCAACTCCTGTGAGCATAGTTG-3′) (Metabion), 2.6 U Expand Long Template Polymerase (Roche, Merck Life Science) and 20 ng of genomic DNA in a final volume of 10 µl. The MJ Research PTC-200 Thermocycler (Bio-Rad) carried out an amplification programme. Cycling conditions were as follows: 94 °C for 2 min to activate polymerase, followed by 15 cycles of 94 °C for 30 s (DNA denaturation), primer annealing at 65 °C for 30 s and amplification at 68 °C for 7 min. Per cycle, 20 s were added to the amplification step. Final elongation was 8 min at 68 °C. The amplification products are one or two bands of the following size (Supplementary Fig. 1a, Online Resource): C9orf72 homozygous wildtype: 3 Kb; C9orf72 heterozygous knockout: 3 Kb and 1 Kb; C9orf72 homozygous knockout: 1 Kb.

Absence of C9orf72 transcript was confirmed by RT-qPCR (Supplementary Fig. 1b, Online Resource). For this RNA was extracted from brain using Trizol (Invitrogen) and purified with PureLink RNA columns (Life Technologies Limited, Paisley, UK). cDNA was produced using the High Capacity cDNA Reverse Transcription Kit (Life Technologies) and RT-qPCR was performed using the Taq Man Gene expression assay (Applied Biosystems, ThermoFisher Scientific) on cDNA triplicates using Universal PCR master mix (Life Technologies) and specific probes for the C9orf72 gene (Mm01216829 m1; Life Technologies) and reference gene β-actin (Mm02619580 g1; Life Technologies). Data were analysed comparatively to control via the ∆∆Ct method [47]. Absence of C9orf72 protein was confirmed on immunoblots (Supplementary Fig. 1c, Online Resource) of protein lysates of C9orf72-WT and C9orf72-KO mouse brains as described previously [76]. Briefly, brains were powdered in liquid nitrogen, homogenised by sonication in ice-cold homogenization buffer (Tris HCl, pH 8, 50 mM, NaCl 150 mM, EGTA pH 8.5 mM, MgCl2 1.5 mM, Triton X-100 1%, anhydrous glycerol 10%, phosphatase and protease inhibitor cocktail; Roche) and centrifuged at 13,000 rpm for 15 min at 4 °C. Hippocampi were homogenised using a Teflon potter in ice-cold homogenization buffer (Tris HCl, pH 8, 50 mM, NaCl 150 mM, EGTA pH 8.5 mM, MgCl2 1.5 mM, Triton X-100 1%, anhydrous glycerol 10%, phosphatase and protease inhibitor cocktail; Roche), sonicated and centrifuged at 13,000 rpm for 15 min at 4 °C. Supernatants were collected and stored at − 80 °C.

Procedures involving animals and their care that were conducted at the Mario Negri Institute for Pharmacological Research IRCCS, Milan, Italy adhered to the Mario Negri Institute for Pharmacological Research IRCCS institutional guidelines, that comply with national (D.lgs 26/2014; Authorization n.493/2019-PR issued on July 4, 2019, by Ministry of Health) and Mario Negri Institutional regulations and Policies providing internal authorisation for persons conducting animal experiments (Quality Management System certificate—UNI EN ISO 9001:2008—reg. N° 6121), the NIH Guide for the Care and Use of Laboratory Animals (2011 edition) and EU directives and guidelines (EEC Council Directive 2010/63/UE). All animals were housed under specific pathogen-free (SPF) conditions at a temperature of 22 ± 1 °C, a relative humidity of 55 ± 10% and 12-h light/dark cycle, 5 per cage. Food (standard pellets) and water were supplied ad libitum.

Procedures involving animals performed at the University of Sheffield, Sheffield, UK, were conducted according to the Animal (Scientific Procedures) Act 1986, under a Project Licence reviewed and approved by the University of Sheffield Ethical Review Sub-Committee, and the UK Animal Procedures Committee (London, UK). The UK Home Office code of practice for the housing and care of animals used in scientific procedures was followed. Mice were bred and housed in an SPF environment using a 12 h light/dark cycle, and a standardised room temperature of 21 ˚C. Mice were fed 2018 rodent diet (Harlan, UK) and provided with water ad libitum.

### Immunohistochemistry

12-week-old mice were deeply anaesthetized with ketamine hydrochloride (150 mg/kg) and medetomidine (2 mg/kg) followed by intracardiac perfusion with PBS 0.1 M (pH 7.4) followed by perfusion with 4% paraformaldehyde (PFA, Merck) in PBS 0.1 M. Brains were removed, post-fixed overnight in PBS 0.1 M with 4% PFA followed by dehydration in PBS with 30% sucrose and embedded in cryostat medium OCT (Sakura Finetek, UK Ltd., Thatcham, UK). Brains were frozen in N-pentane at − 45 °C for 3 min, stored at − 80 °C before 30 µm coronal sections were cut on a cryostat. Free floating sections were blocked and permeabilized in PBS 0.01 M with 10% goat serum and 0.2% Triton and incubated overnight at 4 °C with primary antibodies in antibody diluent (PBS 0.01 M with 1% goat serum and 0.2% Triton) before being washed with PBS 0.01 M and incubated for 1 h at room temperature with secondary antibodies in PBS 0.01 M with 1% goat serum. After washing with PBS 0.01 M, sections were mounted on glass slides with coverslips using FluorSaveTM (Calbiochem, VWR International Ltd., Lutterworth, UK). Z-stacks of images were acquired on an Olympus IX81 microscope with a confocal scan unit (FV500, Olympus). Z-stacks of co-stained sections were acquired using the sequential scanning mode on an A1 Nikon confocal running NIS Elements at 40X magnification. For nuclear staining, sections were stained with Hoechst 33258 (1:500, Invitrogen) and images were acquired using an Olympus virtual slide system VS110 at 40 × magnification with VS-ASW software (Olympus) for image processing.

Immunohistochemistry staining of human brain samples was carried out on formalin-fixed, paraffin-embedded blocks of hippocampus and middle frontal gyrus of ALS/FTD patients with hexanucleotide expansions of the C9orf72 gene, non-C9orf72 related FTD patients as well as neurologically healthy controls. These post-mortem tissues were donated to the Sheffield Brain Tissue Bank (SBTB) with the consent of the next of kin. The SBTB Management Board gave ethical approval for this study under the provision to act as a Research Tissue Bank as approved by the Scotland A Research Ethics Committee (Ref 19/SS/0029, IRAS project ID 261271). Human sample information (pathological diagnosis, age, sex and *C9orf72* status) is provided in Table 2. Sections of paraffin-embedded tissue were subjected to heat induced epitope retrieval (pressure cooker) at pH 9. Immunohistochemistry was carried out using standard ABC techniques with diaminobenzidine (DAB) as chromogen (Vectastain Elite ABC-HRP Kits, ImmPACT^®^ DAB EqV Peroxidase (HRP) Substrate, Vector Laboratories Inc., Burlingame, CA, USA) with antibodies to Syn1, SV2 or C9orf72. Stained slides were digitised using the Hamamatsu NanoZoomer XP, visualized by NDP.view2 digital pathology software (Hamamatsu Photonics). Regions of interest from CA3 and CA4 areas were selected for digital image analysis by a qualified neuropathologist (JRH). Analysis was performed blinded to the genotype.

**Table 2:** Demographic, clinical and pathological details of ALS/FTLD, FTLD and control cases

ID	Sex	Age	Clinicopathological diagnosis	*C9orf72* status
MND/ALS and FTLD cases with C9orf72 repeat expansion^a,b^				
118/2001	M	64	MND/ALS-TDP	Mutant
066/2008	F	59	FTLD-MND/ALS	Mutant
083/2010	M	79	MND/ALS-TDP	Mutant
039/2011	M	71	FTLD-MND/ALS	Mutant
053/1996	F	61	MND/ALS-TDP	Mutant
MND/ALS and FTLD cases without C9orf72 repeat expansion^c^				
233/1999	M	71	FTLD-MND/ALS	Wildtype
060/2012	M	78	FTLD-TDP	Wildtype
006/2009	F	58	FTLD-TDP	Wildtype
Neurologically healthy control cases				
005/2007	M	63	Control	Wildtype
085/2007	F	59	Control	Wildtype
070/2007	M	26	Control	Wildtype
098/2007	M	68	Control	Wildtype

^a^Patients presented to an ALS clinic and developed variable FTD-associated behavioural and neuropsychiatric symptoms during the course of their illness

^b^C9orf72 status determined by PCR

^c^C9orf72 status defined by absence of C9orf72 repeat expansion specific neuropathology (stellate inclusions in hippocampus)

### Electron microscopy

12-week-old mice were deeply anaesthetized with ketamine hydrochloride (150 mg/kg) and medetomidine (2 mg/kg) followed by intracardiac perfusion with PBS 0.1 M followed by 2.5% glutaraldehyde (Electron Microscopy Sciences, Hatfield, PA, USA) and 4% PFA in PBS 0.1 M. Brains were removed, post-fixed overnight in 2.5% glutaraldehyde and 4% PFA in PBS 0.1 M and washed 3–4 times for 1 h at 4 °C. A ~ 1 mm^3^ block containing the hippocampus was micro-dissected out from each whole brain before being treated with osmium tetroxide (1% in 0.1 M PBS) for 30 min. Samples were dehydrated through an ascending series of ethanol and propylene oxide, before embedding in Durcupan resin. During dehydration, sections were treated with uranyl acetate (1% in 70% ethanol) for 40 min. Durcupan resin was polymerised for 48 h in an oven set to 56 °C. Smaller regions of interest containing the CA3 region of hippocampus were identified and then cut from Durcupan-embedded sections before being mounted on Durcupan blocks and cutting ultra-thin sections (~ 70 nm) on an ultracut microtome (Leica) with an Ultra 45 Diamond Knife (Diatome, Hatfield, PA, USA). Sections were collected on formvar-coated slot grids and stained with lead citrate before imaging on a JEOL transmission electron microscope equipped with a Gatan digital camera (Jeol UK Ltd., Welwyn Garden City, UK).

### Image analysis

Image analysis was performed using ImageJ/Fiji (http://imagej.nih.gov/ij/) [63] and CellProfiler-4.1.3 software [52].

PLA fluorescence intensities in EGFP-positive HEK293 cells were measured and the corrected total cell fluorescence (CTCF, arbitrary units, a.u.) was calculated as integrated density—(cell area × mean background fluorescence) using Fiji (http://imagej.nih.gov/ij/).

PLA signal in hippocampal neurons were analysed by counting the number of epifluorescence PLA signal spots detected per image using the ‘find maxima’ routine included in Fiji. The number of spots detected was normalized to the total mean pixel grey value of the synaptophysin epifluorescence intensity of the same image (norm PLA). Synaptophysin staining accounts for variability in neuron culture density across coverslips.

Synapse densities and properties of pre- and postsynaptic staining in 4-colour confocal images of cultured hippocampal neurons were analysed using an automated analysis pipeline in CellProfiler-4.1.3 [52]. Briefly, the dendritic marker MAP2 was used to identify neurites after feature enhancement using the Tubeness method and exclusion of cell body staining. To quantify neurite length, we binarized and skeletonised the enhanced neurite images and measured the skeleton intensity. To detect synapses, images of post- and presynapse markers were smoothed, enhanced using a white tophat filter and thresholded. Of the detected postsynapses only those that mapped to neurites were retained. Synapses were tallied when presynapses overlapped with postsynapses. For each image, measures of synapse density (synapse count/dendrite length) and mean values of pre- and postsynaptic parameters such as area size and intensity of pre- and postsynaptic markers of the identified synapses were exported to Microsoft Excel (Microsoft Corporation, Redmond, WA, USA).

To quantify immune staining of brain sections of C9orf72-WT and heterozygous and homozygous C9orf72-KO mice, confocal imaging stacks were analysed using Fiji and CellProfiler. Stacks were background subtracted and Z-projected (Sum of slices). For single stain images (Fig. 4), regions of interest (ROI) containing the mossy fibre area (hilus of the dentate gyrus (CA4) and the CA3/CA2 regions) were outlined manually by the operator while being blinded to the genotype. For higher magnification dual stain images (Fig. 5), regions of interest (ROI) containing hilus of the dentate gyrus (CA4) were selected. Projections and image masks created from the ROIs were imported in CellProfiler for analysis. For single stain images (Fig. 4) the area fraction and fluorescence intensity was determined. Positive signal within the masked mossy fibre area was determined by Robust Background thresholding (mean + 1 standard deviation). Fluorescence intensity was measured as the mean grey level of the thresholded area. For dual stained sections (Fig. 5), images of post- and presynapse markers were smoothed and enhanced using a white tophat filter and thresholded using the average threshold determined over all C9orf72-WT sections using the Robust Background thresholding method (mean + 1 standard deviation). Synapses were defined as postsynaptic spots that co-occurred with presynaptic spots. The number of synapses were normalised to the size of the region of interest to yield synaptic density.

On images of nuclear staining of the entire hippocampus, ROIs of the dentate gyrus were outlined manually and analysed blinded to the genotype to calculate the nuclei density (nuclei/mm^2^) of the dentate gyrus using Fiji.

To quantify DAB staining in C9ALS/FTD or FTD patient and healthy control brain sections, RGB images were analysed with Fiji using an automated colour threshold using the triangle method followed by particle analysis to determine the percentage of total image area positive for DAB staining. All analysis was performed blinded to the sample ID and disease status.

Individual synaptic profiles were identified in EM images based on the presence of clear pre- and postsynaptic elements, synaptic vesicles within the presynaptic nerve terminal, and a clear postsynaptic density [25, 38]. Synaptic vesicle counts were performed as described previously [24]. Briefly, vesicles were defined by the presence of a clear lumen, unbroken membranes, and a diameter of ∼ 50 nm. Pre-synaptic active zones were identified by their position opposite a postsynaptic density. Numbers of docked vesicles were estimated by measuring vesicles within a 125 nm radius of the presynaptic membrane. Analysis of TEM images was performed blind to the genotype of each sample.

### Statistical analysis

Data are presented as bar charts plots with mean ± SEM or box and whisker plots where the box extends from the 25th to 75th percentiles and the whiskers show minimum and maximum; the line in the middle of the box is plotted at the median. All graphs were generated using Prism 9 software (GraphPad Software LLC., San Diego, CA, USA).

Calculations and statistical analysis were performed using Excel (Microsoft, Redmont, WA, USA) and Prism 9 software. Statistical significance between experimental groups was determined by one-way analyses of variance (ANOVA), paired or unpaired two-tailed *t* test, or one sample *t* and Wilcoxon tests, according to the data structure and distribution. Multiple comparisons following ANOVA were performed using "Tukey's multiple comparisons test". Sample sizes and further details can be found in the figure legends.

## Results

### C9orf72 interacts with synapsin

To identify novel interactors of C9orf72, we screened a human full-length cDNA yeast two-hybrid (Y2H) library containing approximately 15,000 ORF clones using the long isoform of human C9orf72 (C9orf72L) [17] as bait. The screen yielded 39 positive interacting clones. Thirty-four of these encoded full-length synapsin-3 (Syn3). The other 5 encoded another novel interacting protein. Therefore, this screen reproducibly revealed a strong interaction of C9orf72 with the presynaptic protein Syn3. Syn3 is a member of the synapsin protein family that in addition to Syn3 includes synapsin-1 (Syn1) and synapsin-2 (Syn2). The synapsin proteins are encoded by three distinct genes, *SYN1*, *SYN2* and *SYN3,* and alternative splicing generates a and b isoforms of Syn1 and Syn2 and isoforms a-f of Syn3 [49]. Synapsins are peripheral membrane proteins found in all presynaptic terminals where they bind to synaptic vesicles and regulate their dynamics [70].

**Fig. 1:**
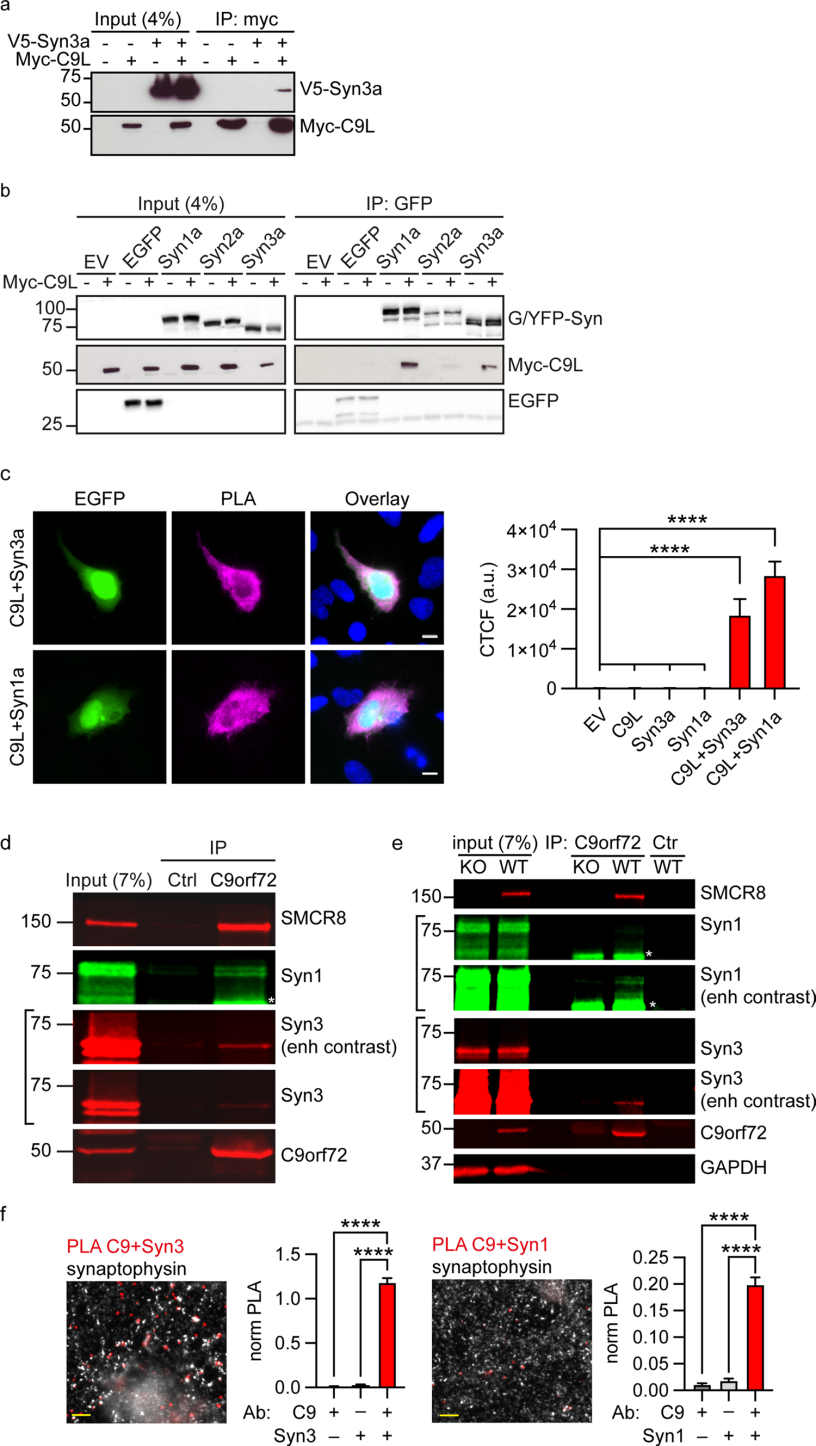
Synapsin family proteins are novel binding partners of C9orf72. **a** Lysates of HEK293 cells co-transfected with Myc-C9orf72L (Myc-C9L) and either empty vector (EV) or V5-tagged Synapsin-3a (V5-Syn3a) were subjected to immunoprecipitation using an anti-Myc antibody. Immune pellets were probed for Myc-C9orf72L and V5-Syn3a on immunoblots. **b** Lysates of HEK293 cells co-transfected with Myc-C9L and either EV, EGFP or EGFP-Syn1a, YFP-Syn2a, EGFP-Syn3a were subjected to immunoprecipitation using an anti-GFP antibody. Immune pellets were probed for GFP/YFP and Myc-C9orf72L on immunoblots. **c** HEK293 cells were co-transfected with either Myc-C9L + V5-Syn3a or with Myc-C9L + V5-Syn1a. Transfections were laced with EGFP (green) to identify transfected cells. Cells were fixed and immunostained with both anti-V5 and anti-Myc antibodies and processed for PLA (PLA, magenta), nuclear staining with Hoechst (blue). Images are representative of the individual channels and their overlay. Scale bar 10 μm. Intensity of PLA signals per cell was analysed as corrected total cellular fluorescence (CTCF); data are presented as mean ± SEM; *n* (cells analysed) EV = 107, C9L = 92, Syn3a = 113, Syn1a = 79, C9L + Syn3a = 129, C9L + Syn1a = 109 from three or 4 replicate experiments. Statistical significance was determined by one-way ANOVA with Tukey’s multiple comparisons test, ****P < 0.0001. Images showing the single transfection controls are shown in Supplementary Fig. 2, Online Resource. **d** Lysates of synaptosomes prepared from 12-week-old mouse brains were subjected to immunoprecipitation using an anti-C9orf72 antibody or an irrelevant antibody (Ctrl) raised in the same species as the C9orf72 antibody. Immune pellets were probed for endogenous SMCR8, C9orf72, Syn3 and Syn1 (* indicates a nonspecific band; enh, enhanced). **e** Lysates of synaptosomes prepared from 12-week-old C9orf72-WT and C9orf72-KO mouse brains were subjected to immunoprecipitation using an anti-C9orf72 antibody or a control antibody (M2). Immune pellets were probed for endogenous SMCR8, C9orf72, Syn3 and Syn1 (* indicates a nonspecific band; enh, enhanced). **f** 12DIV primary rat hippocampal neurons were fixed and immunostained with pairs of antibodies against C9orf72 and Syn3 (C9 + Syn3) or C9orf72 and Syn1 (C9 + Syn1) together with an antibody against synaptophysin and processed for PLA. Overlay PLA signals (red) between C9 + Syn3 or C9 + Syn1 and synaptophysin (white). Scale bar 5 μm. For quantification, the number of PLA spots per image was normalised (norm PLA) to the mean grey intensity of the synaptophysin image. Data are presented as mean ± SEM; *n* (images analysed) C9 = 14, Syn1 = 14, Syn3 = 15, C9 + Syn1 = 59, C9 + Syn3 = 62 from two or three replicate experiments. Statistical significance was determined by one-way ANOVA with Tukey’s multiple comparisons test, ****P < 0.0001. Single antibody controls are shown in Supplementary Fig. 2, Online Resource

To confirm the interaction between C9orf72 and synapsin, we first performed co-immunoprecipitation assays of transfected proteins in HEK293 cells. In agreement with the Y2H interaction, V5-tagged Syn3a (V5-Syn3a) co-immunoprecipitated with Myc-tagged C9orf72L (Myc-C9orf72L) from V5-Syn3a and Myc-C9orf72L co-transfected cells but not from cells transfected with Myc-C9orf72L or V5-Syn3a alone (Fig. 1a). To determine whether the interaction between C9orf72 and synapsin was specific for the Syn3a isoform, we co-transfected HEK293 cells with Myc-C9orf72L and either empty EGFP vector, EGFP-tagged Syn1a or Syn3a, or YFP-tagged Syn2a. EGFP/YFP-synapsin was immunoprecipitated and the immune pellets were probed for EGFP/YFP and Myc. C9orf72 specifically co-immunoprecipitated with all three synapsin isoforms in these assays, albeit less efficiently with Syn2a (Fig. 1b).

C9orf72 is known to form a complex with SMCR8 [64]. Since there is no SMCR8 ortholog in yeast, the Y2H data above show that interaction of C9orf72 and Syn3 does not require SMCR8. To further investigate a possible role of SMCR8 in the interaction of C9orf72 with synapsin we immunoprecipitated transfected SMCR8 carrying an mycDDK tag (SMCR8-mycDDK) from CRISPR/Cas9 C9orf72 knockout (KO) HEK293 cells (Supplementary Fig. 2a, Online Resource; Supplementary Materials and Methods, Online Resource) and probed the immune pellet for co-transfected V5-Syn3a. SMCR8 did not co-immunoprecipitate V5-Syn3a in absence of C9orf72. In contrast, when Myc-C9orf72L was co-transfected with SMCR8-mycDDK and V5-Syn3a, SMCR8 efficiently co-immunoprecipitated V5-Syn3a (Supplementary Fig. 2b, Online Resource). Hence synapsin interacts with the C9orf72/SMCR8 complex by interaction with C9orf72 but not SMCR8.

We next investigated the C9orf72/synapsin interaction in its cellular context using proximity ligation assays (PLA). Similar to conventional immunofluorescence, PLA uses pairs of primary antibodies that selectively recognise putative interacting proteins in fixed cells but the secondary antibodies used are linked to complementary oligonucleotide sequences (PLA probes) that generate a fluorescent ligation product when the two probes are in close proximity (< 40 nm); each signal corresponds to an interacting protein pair [69]. Using antibodies to the V5 and Myc tags, respectively, we observed proximity signals in all cells co-transfected with Myc-C9orf72L and V5-Syn1a or V5-Syn3a whereas very few signals were detected in empty vector or single transfected controls (transfections were laced with EGFP to identify transfected cells; Fig. 1c, Supplementary Fig. 3, Online Resource). Quantification of the background corrected PLA fluorescence signal showed that PLA intensities were significantly higher in cells co-expressing Myc-C9orf72L and V5-Syn3a or Myc-C9orf72L and V5-Syn1a compared to the controls (Fig. 1c, Supplementary Fig. 3, Online Resource). Thus, C9orf72 interacts with synapsin in situ.

Given the presynaptic localisation of C9orf72 and synapsin [21, 70] these data indicated that C9orf72 might interact with synapsin at synapses. Both synapsin and C9orf72 are highly expressed in the hippocampus, particularly within the mossy fibre system [21, 42, 53, 68, 84]. We therefore co-immunostained 12DIV hippocampal neuron cultures using KO verified antibodies to C9orf72, Syn1 or Syn3 (Table 1), and the synaptic vesicle protein synaptophysin. Consistent with interaction of C9orf72 with synapsin at synapses, a proportion of endogenous C9orf72 co-localised with Syn3 and Syn1 at synapses co-labelled with synaptophysin (Supplementary Fig. 4a, b, Online Resource). Furthermore, biochemical analysis of synaptosomes (biochemical preparations of “pinched-off” isolated nerve terminals [29]) showed that C9orf72 is enriched in synaptosomal fractions (Supplementary Fig. 4c, Online Resource). To confirm the interaction of endogenous C9orf72 and synapsin at synapses we performed co-immunoprecipitation assays from synaptosome fractions prepared from 12-week-old C9orf72 mouse brains. Both endogenous Syn3 and Syn1 co-immunoprecipitated with C9orf72 but not when a control antibody (Ctrl) was used (Fig. 1d, e). We further validated the endogenous interaction of C9orf72 and synapsin in C9orf72 wildtype (C9orf72-WT) and C9orf72 homozygous KO (C9orf72-KO) mouse brains. Syn3 and Syn1 were only co-immunoprecipitated with C9orf72 from wildtype but not from C9orf72-KO synaptosome fractions (Fig. 1e). SMCR8 was also detected in the co-immunoprecipitation (Fig. 1d, e). Finally, we confirmed C9orf72/synapsin interactions in 12DIV rat hippocampal neurons in situ by PLA. Using C9orf72/Syn3 or C9orf72/Syn1 antibody pairs we observed discrete PLA signals (Fig. 1f, Supplementary Fig. 5, Online Resource) whereas PLA signals were largely absent in the single antibody control samples (Supplementary Fig. 5, Online Resource). Consistent with the co-localisation results, a fraction of the C9orf72/synapsin PLA spots co-stained for the presynaptic marker synaptophysin indicating a direct interaction of C9orf72 with synapsin in synapses (Fig. 1f, Supplementary Fig. 5, Online Resource). Quantification of total number of PLA spots per image, normalised to the mean synaptophysin intensity, showed that the PLA signal was significantly higher using C9orf72/Syn3 or C9orf72/Syn1 antibody pairs compared to single antibody controls (Fig. 1f, Supplementary Fig. 5, Online Resource).

Together these data identify synapsin family proteins as novel binding partners of C9orf72 at synapses.

### The N-terminal longin domain of C9orf72 interacts with the conserved C domain of synapsin

**Fig. 2:**
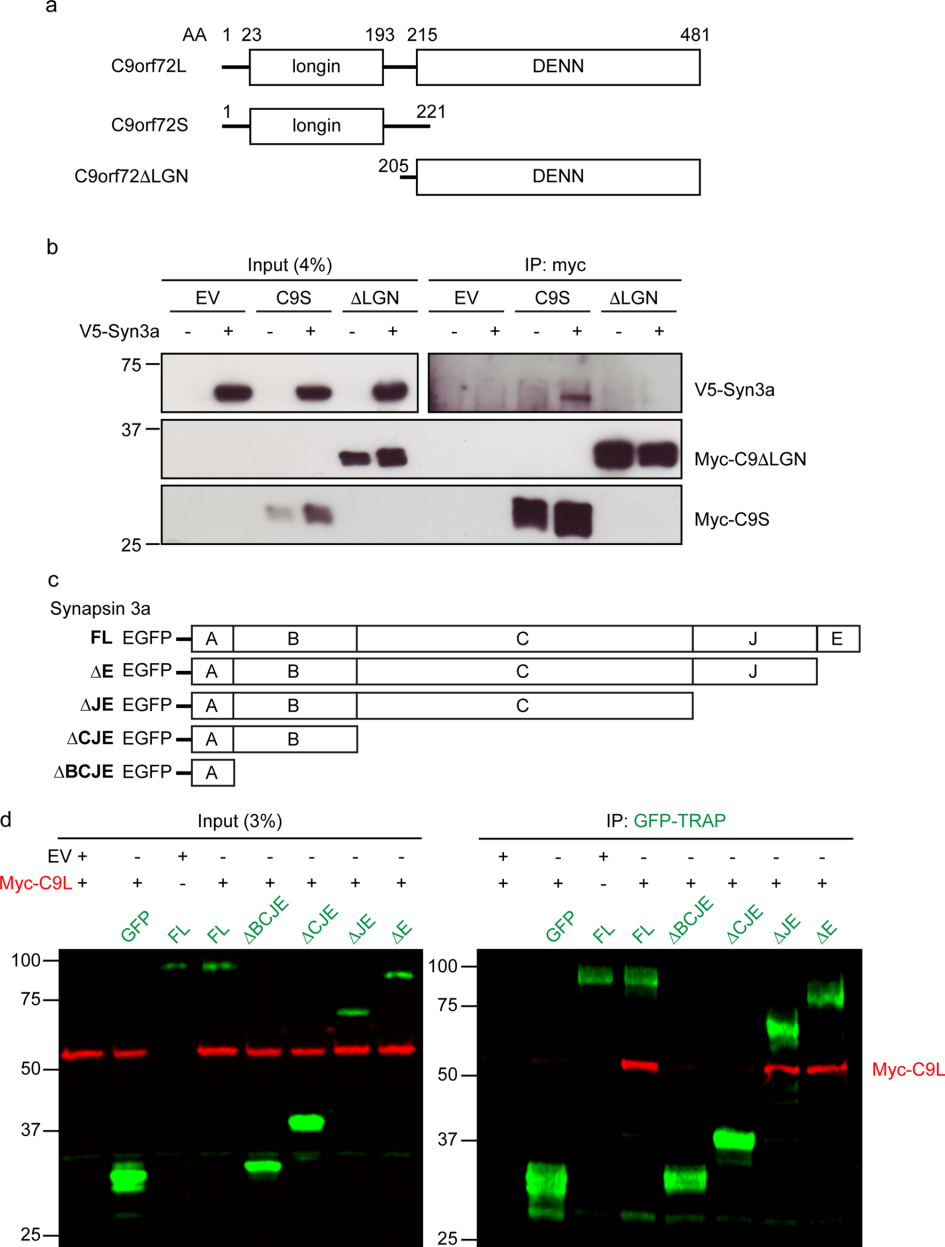
The N-terminal longin domain of C9orf72 interacts with the C domain of synapsin. **a** Schematic representation of human C9orf72L, C9orf72S and the truncated C9orf72ΔLGN protein with their corresponding domain boundaries. **b** Cell lysates of HEK293 cells co-transfected with V5-Syn3a and either empty vector (EV), Myc-C9orf72S (C9S) or Myc-C9orf72ΔLGN (ΔLGN) were subjected to immunoprecipitation using an anti-Myc antibody. Immune pellets were probed for Myc-C9orf72 and V5-Syn3a on immunoblots. **c** Schematic representation of full-length EGFP-Syn3a (FL) and truncated EGFP-Syn3a proteins with removed domains indicated (∆E, ∆JE, ∆CJE, ∆BCJE). **d** HEK293 cells were co-transfected with either empty vector (EV) or Myc-C9orf72L (Myc-C9L) together with EGFP (GFP), full-length EGFP-Syn3a (FL) or truncated (∆E, ∆JE, ∆CJE, ∆BCJE) EGFP-Syn3a. Lysates were subjected to immunoprecipitation using GFP-TRAP beads. Immune pellets of input and IP samples were probed for Myc-C9orf72L (red) and EGFP or EGFP-Syn3a (green)

C9orf72 is a member of the Differentially Expressed in Normal and Neoplastic cells (DENN) protein family. C9orf72L contains an N-terminal longin domain (also referred to as uDENN; for *upstream* DENN) and a C-terminal DENN domain; C9orf72S encompasses only the N-terminal longin domain (Fig. 2a) [45, 74, 85]. To determine which domains of C9orf72 interact with synapsin, we co-transfected HEK293 cells with V5-Syn3a and either Myc-C9orf72S or a Myc-tagged N-terminal truncated C9orf72L construct lacking the longin domain (Myc-C9orf72ΔLGN) and immunoprecipitated Myc-C9orf72. V5-Syn3a specifically co-immunoprecipitated with Myc-C9orf72S but not Myc-C9orf72ΔLGN (Fig. 2b). Therefore, synapsin interacts with the N-terminal longin domain of C9orf72.

All synapsin isoforms share three conserved N-terminal domains (domains A, B and C), but differ at their C-terminus [70]. The interaction of C9orf72 with all synapsin isoforms (Fig. 1b) suggested that binding might be mediated by the conserved A, B, and C domains common to all isoforms. To test this directly, we generated a number of C-terminal truncated EGFP-tagged Syn3a constructs by sequentially removing domain E (Syn3aΔE), domains E and J (Syn3aΔJE), domains E, J, and C (Syn3aΔCJE), and domains E, J, C, and B (Syn3aΔBCJE) from full-length Syn3a (Fig. 2c). Removal of domain E (Syn3aΔE) or domains E and J (Syn3aΔJE) did not affect co-immunoprecipitation of Myc-C9orf72L from transfected HEK293 cells whereas removal of domain C (Syn3aΔCJE and Syn3aΔBCJE) completely abrogated the interaction of C9orf72 with synapsin in these assays (Fig. 2d).

Thus, synapsin interacts via its conserved C domain with the N-terminal longin domain of C9orf72.

### C9orf72 haploinsufficiency impairs excitatory synapses

Dysfunction of synapses is a common feature in many neurodegenerative diseases, including C9ALS/FTD [30, 31, 56, 57]. Given the interaction of C9orf72 with synapsin at synapses (Fig. 1), we reasoned that C9orf72 haploinsufficiency might affect synapses. To investigate this possibility, we quantified synaptic density, size and synaptic protein expression levels in 12DIV hippocampal neuron cultures in which C9orf72 expression was knocked down by lentiviral C9orf72-targeting miRNA (miRNA-C9) [80]. RT-qPCR showed that compared to cultures treated with non-targeting control miRNA (miRNA-NTC), C9orf72 mRNA levels were reduced by approximately 65 ± 4% (mean ± SEM) in cultures treated with miRNA-C9 (Supplementary Fig. 6a, Online Resource). Analysis by immunoblot demonstrated a decrease of approximately 49 ± 7% (mean ± SEM) in C9orf72 protein levels in hippocampal neuron cultures treated with miRNA-C9 (Supplementary Fig. 6b, Online Resource). Thus, C9orf72 knockdown using lentiviral miRNA-C9 mimics the C9orf72 haploinsufficiency reported in human C9ALS/FTD cases.

**Fig. 3:**
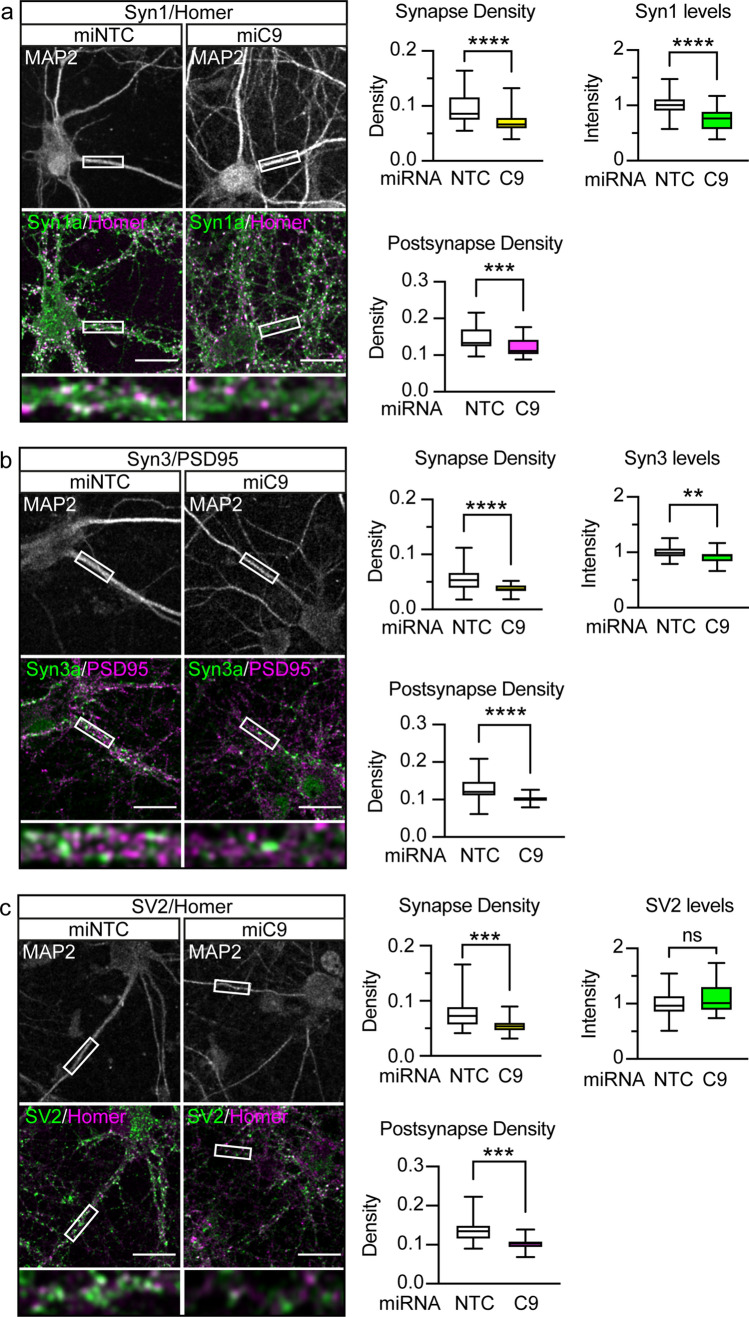
C9orf72 haploinsufficiency reduces the number of excitatory synapses. Primary rat hippocampal neurons were transduced with EmGFP non-targeting control miRNA (miNTC) or C9orf72 miRNA (miC9) lentivirus at 5DIV. Neurons were immunostained at 12DIV for the dendritic marker MAP2 (white) and pre- (green) and postsynaptic (magenta) pairs: **a** Syn1 and Homer, **b** Syn3 and PSD95, **c** SV2 and Homer. Representative confocal images are shown. Boxes denote zoom area, note: co-localisation of pre- (green) and postsynaptic marker (magenta) appears white. Scale bar: 10 μm. Synapse density and the density of postsynapses was quantified per image for each staining pair and are presented as box and whisker plots. The mean intensity of presynaptic staining within detected synapses was quantified per image and is presented as box and whisker plots. **a** Syn1/Homer with n (images analysed) miRNA NTC = 41, miRNA C9 = 43 from 4 replicate experiments, **b** Syn3/PSD95, with n (images analysed) miRNA NTC = 30, miRNA C9 = 30 from three replicate experiments, **c** SV2/Homer with *n* (images analysed) miRNA NTC = 29, miRNA C9 *n* = 30 from three replicate experiments. Statistical significance was determined by unpaired two-tailed *t* test, ns (not significant), *P < 0.05, **P < 0.01, ***P < 0.001, ****P < 0.0001

We first examined the effect of C9orf72 knockdown on excitatory synapses by co-staining of presynaptic Syn1 or Syn3 with the excitatory postsynaptic markers Homer or PSD95, respectively (Fig. 3a, b). Synapses were identified by co-occurrence of pre- and postsynaptic markers and quantified relative to the size of the dendritic compartment labelled with microtubule-associated protein 2 (MAP2) to yield the synaptic density. To ensure bona-fide synapses were counted, only synapses with postsynaptic staining within the dendritic compartment were considered. Analysis of synaptic density showed that knockdown of C9orf72 significantly reduced the number of Syn1 and Syn3-positive excitatory synapses by 25.22 ± 2.899% and 33.66 ± 5.183%, respectively (mean ± SEM; Fig. 3a, b). Further morphological analysis showed that knockdown of C9orf72 had no effect on the size of excitatory presynapses (Table 3).

**Table 3:** C9orf72 knockdown does not affect excitatory presynaptic area

Staining	miRNA-NTC			miRNA-C9orf72			*P* value
	Area	SD	*n*	Area	SD	*n*	
Syn1/Homer	16.06	2.44	41	15.21	1.85	43	0.0766 (ns)
Syn3/PSD95	14.38	1.73	30	14.39	1.43	30	0.9667 (ns)
SV2/Homer	13.28	1.36	29	13.88	1.95	30	0.1747 (ns)

The area of excitatory presynapses was quantified in 12DIV primary rat hippocampal neurons that had been transduced with EmGFP non-targeting control miRNA (NTC) or C9orf72 miRNA. Presynapses were labelled by immunostaining of Syn1, Syn3 or SV2 and co-stained with the excitatory postsynaptic markers Homer or PSD95 as indicated to allow identification of excitatory synapses. Data are presented as the mean and standard deviation (SD) of n images obtained from three (Syn3/PSD95 and SV2/Homer) or 4 (Syn1/Homer) independent experiments. Statistical significance was determined by unpaired two-tailed *t* test

*ns* not significant

To examine whether C9orf72 haploinsufficiency impacted on the levels of synapsin at synapses we assessed protein levels in the remaining synapses. C9orf72 knockdown caused significant reductions in Syn1 and Syn3 levels at synapses (Fig. 3a, b). This reduction in synaptic Syn1 and Syn3 levels was not due to a global effect on synapsin expression because immunoblot of total cell lysates did not show differences between the groups (Supplementary Fig. 6c, d, Online Resource).

The above data show that knockdown of C9orf72 reduced the number of synapsin-positive excitatory synapses and reduced synapsin staining in remaining excitatory synapses. To distinguish whether the loss of synapsin-positive synapses reflected a genuine reduction in the number of synapses or whether the effect observed was instead due to failure to detect presynapses devoid of synapsin, we utilised SV2, a membrane proteoglycan specifically found in the secretory vesicles of neuronal cells [77], as an independent presynaptic marker to assess synapses in C9orf72-haploinsufficient cultures. Co-staining of SV2 with Homer revealed that the density of SV2-positive synapses was reduced by 27.36 ± 8.155% in hippocampal neuron cultures treated with miRNA-C9 (Fig. 3c), similar to the observed decrease in synapsin-positive synapses. In contrast to synapsin, there was no change in the levels of SV2 in the remaining excitatory synapses (Fig. 3c). Thus, these data indicate that C9orf72 knockdown causes a genuine reduction in excitatory synapses in these cultures.

To further dissect the relative contribution of synapse loss versus reduced synapsin levels in our cultures, we analysed the density of postsynapses. We reasoned that loss of synapses would be reflected in an equivalent reduction in postsynapses, whereas in case of presynapses being merely devoid of synapsin and therefore escaping detection, postsynaptic densities would be unaffected. Analysis of postsynapse density showed that the 25.22 ± 2.899% and 33.66 ± 5.183% reduction in Syn1 and Syn3-positive synapses was partially matched by a 15.92 ± 2.055% (*p* = 0.0187, paired *t* test, *n* = 4 experiments), and 22.78 ± 3.311% (*p* = 0.0295, paired *t* test, *n* = 3 experiments) reduction in postsynapses, respectively (Fig. 3a, b). In contrast the decrease in SV2-positive synapses of 27.36 ± 8.155% was completely matched by a 23.37 ± 6.526% decrease in postsynapses (*p* = 0.2793, paired *t* test, *n* = 3 experiments, Fig. 3c). Thus, these data indicate that the reduction in the number of synapsin-positive synapses observed after C9orf72 knockdown is caused in part by a loss of excitatory synapses and in part by non-detection of synapses devoid of synapsin.

A recent report showed that C9orf72 deficiency led to enhanced microglia-mediated synaptic pruning and loss of synapses in the motor cortex of aged C9orf72-KO mice [43]. To investigate whether the changes in synapse density we observed in our cultures involved microglial pruning, we co-stained 12DIV hippocampal neuron cultures with a microglial marker, Iba1, a neuronal marker, beta-III tubulin, and a marker for non-neuronal cells, vimentin. No Iba1-positive microglia were observed in our hippocampal neuron cultures which are grown under conditions that are formulated specifically for neuronal cell requirements (Supplementary Fig. 7a, Online Resource). As a control we set up hippocampal cultures under specific conditions that support non-neuronal cells and confirmed that these contained Iba1-positive cells (Supplementary Fig. 7a, Online Resource). Thus, the reduction in the number of synapses observed after C9orf72 knockdown in our hippocampal neurons cultures is not caused by microglia-mediated elimination of synapses.

Changes in the ratio of functional excitatory and inhibitory synapses can affect the balance between excitatory and inhibitory neurotransmission. Disturbance of this equilibrium has emerged as a contributor to neurological disorders and neurodegenerative diseases, including ALS/FTD [23, 78, 83]. To determine if the effect of C9orf72 haploinsufficiency was specific for excitatory synapses, we labelled inhibitory synapses using antibodies to presynaptic Syn3 or the vesicular GABA transporter (VGAT) in combination with gephyrin, a postsynaptic scaffold in inhibitory glycinergic and GABAergic synapses [41]. In contrast to excitatory synapses, C9orf72 knockdown had no effect on the density of either Syn3- or VGAT-positive inhibitory synapses (Supplementary Fig. 7b, c, Online Resource). Similarly, there was no effect on the size of inhibitory presynapses (Supplementary Table 1, Online Resource) or levels of Syn3 in inhibitory presynapses, but there was a small reduction in VGAT levels (Supplementary Fig. 7b, c, Online Resource). Finally, knockdown of C9orf72 had no effect on the density of gephyrin-positive postsynapses (Supplementary Fig. 7b, c, Online Resource). Thus, C9orf72 haploinsufficiency selectively affects excitatory synapses.

### Loss of C9orf72 impairs excitatory synapses in the hippocampus in vivo

The data presented above show that C9orf72 knockdown in dissociated hippocampal neuron cultures reduces the density of excitatory synapses. Furthermore, the levels of Syn1 and Syn3 were markedly reduced at excitatory synapses after knockdown of C9orf72 (Fig. 3). Even though embryonic hippocampal cultures are a well-characterised and commonly utilised model to study synapses, they lack the complexity and the tissue specific context of neurons within the intact brain. We therefore analysed the effects of loss of C9orf72 in the hippocampus of C9orf72-KO mice.

**Fig. 4:**
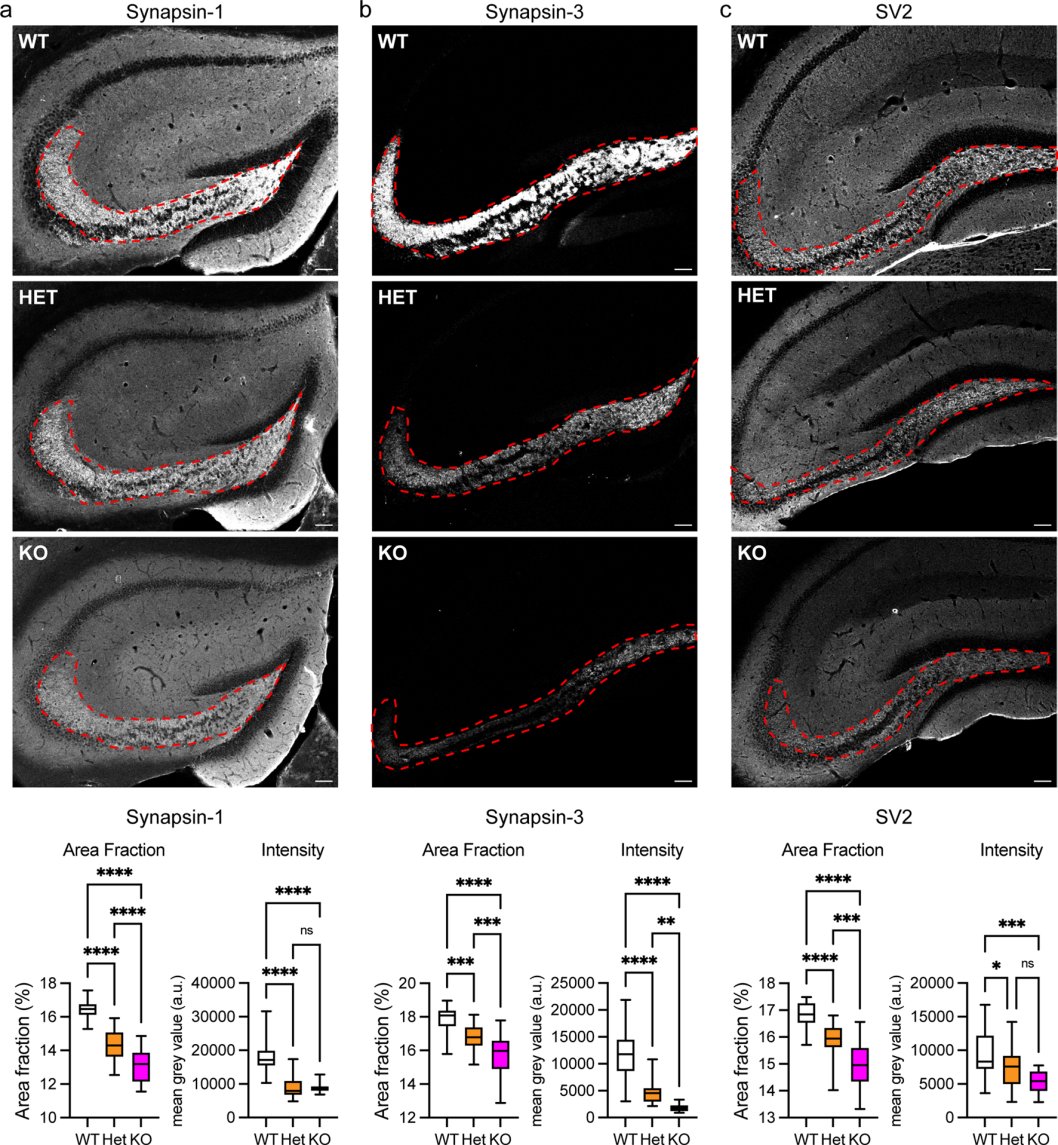
Loss of C9orf72 reduces levels of synapsin in the hippocampus in vivo*.* Representative images of hippocampal sections of 12-week-old C9orf72-WT, C9orf72-HET and C9orf72-KO mice stained for **a** Syn1, **b** Syn3 or **c** SV2. Syn1, Syn3, and SV2 levels in the mossy fibre area (outlined) were quantified as the area fraction of positive staining (Area Fraction) and the mean fluorescence intensity level (Intensity) within the outlined area was determined per section. Scale bar 100 µm. Data are presented as box and whisker plots; **a** Syn1, *n* (sections analysed) WT = 26, HET = 26, KO = 27 from 5 animals/genotype; **b** Syn3, *n* (sections analysed) WT = 30, HET = 28, KO = 26 from 5 animals/genotype; **c** SV2, *n* (sections analysed) WT = 26 HET = 24 and KO = 16 from 5 animals/genotype. Statistical significance was determined by one-way ANOVA with Tukey’s multiple comparisons test, ns (not significant), *P < 0.05, **P < 0.01, ***P < 0.001, ****P < 0.0001

To investigate if loss of C9orf72 affected synapses in the hippocampus in vivo, we immunostained coronal sections of the dorsal hippocampus of 12-week-old C9orf72-WT, heterozygous C9orf72-KO (C9orf72-HET) and homozygous C9orf72-KO mice using antibodies to Syn1, Syn3 or the non-synapsin family presynaptic vesicle marker SV2. Staining for Syn1 and Syn3 was particularly prominent in the hippocampal mossy fibre system, namely the hilus area of the dentate gyrus (CA4) and the CA3/CA2 region (Fig. 4a–c), where also C9orf72 expression is pronounced [21, 42]. To assess if synapsin-positive synapses were affected by C9orf72 knockout we first determined the area fraction positive for Syn1 or Syn3 staining within this area of the hippocampus. For both Syn1 and Syn3, the area fraction of staining showed a significant, gene dosage-dependent reduction in C9orf72-HET and C9orf72-KO compared to C9orf72-WT controls (Fig. 4a, b). Quantification of protein levels by measuring the fluorescence intensity within this area showed a significant, gene dosage-dependent reduction in Syn3 levels in C9orf72-HET and C9orf72-KO (Fig. 4a, b). Syn1 levels were significantly reduced compared to C9orf72-WT controls but to the same extend in C9orf72-HET and C9orf72-KO. The reduction in synapsin levels was not due to a general effect on gene expression since immunoblot analysis of whole brain and hippocampal lysates did not show a difference between C9orf72-WT and C9orf72-KO mice (Supplementary Fig. 8a, Online Resource).

**Fig. 5:**
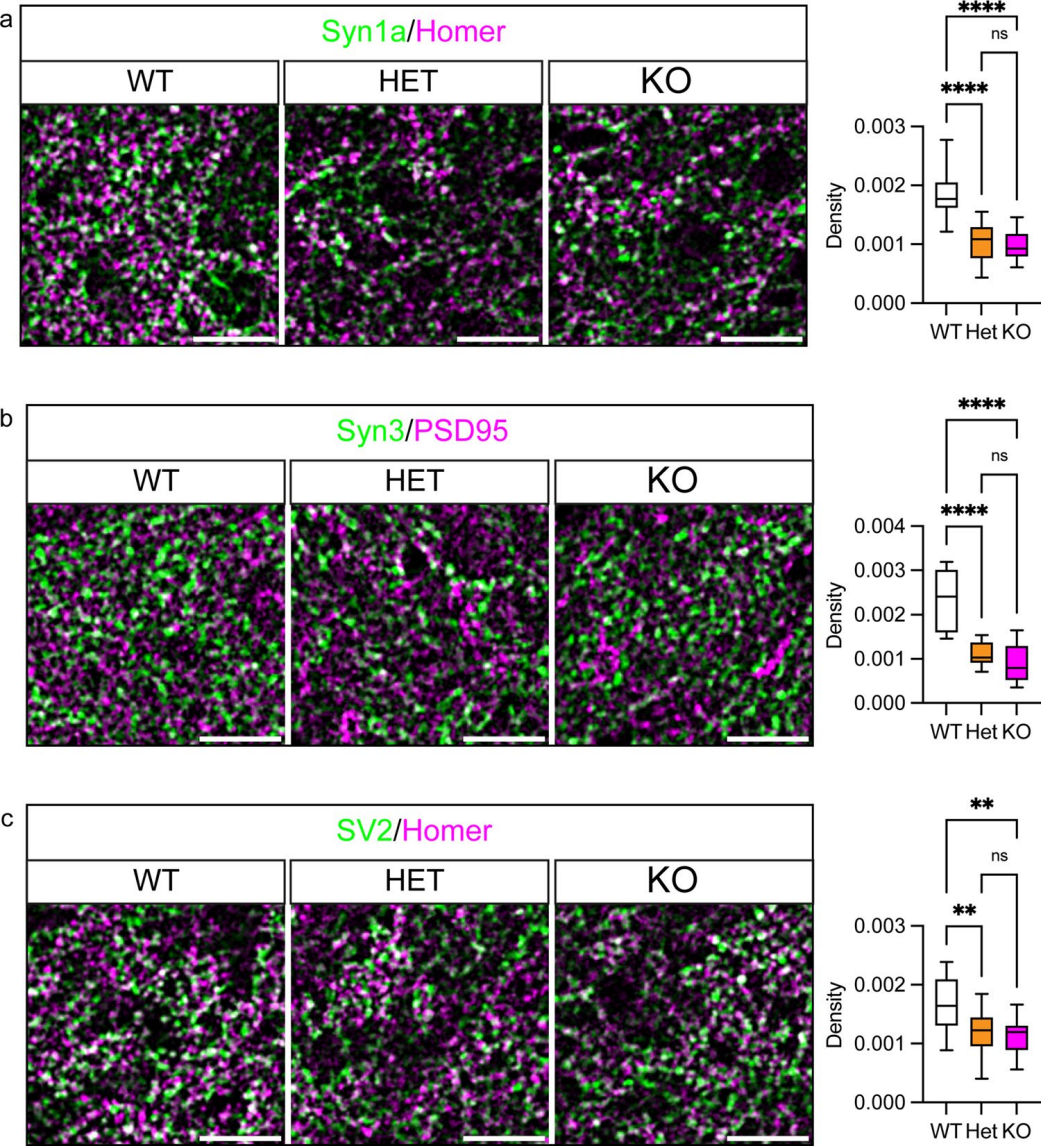
Loss of C9orf72 reduces synaptic density in the hippocampus in vivo*.* Representative enhanced images showing **a** Syn1/Homer (green/magenta), **b** Syn3/PSD95 (green/magenta) or **c** SV2/Homer (green/magenta) pre- and postsynaptic marker pairs in the hilus of the dentate gyrus (CA4) region of the hippocampus of 12-week-old C9orf72-WT, C9orf72-HET and C9orf72-KO mice. Scale bar 20 µm. Synaptic density (density) was determined from the co-occurrence of pre- and postsynaptic marker pairs (white) in the hilus of the dentate gyrus (CA4) region. Data are presented as box and whisker plots; **a** Syn1/Homer, *n* (sections analysed) WT = 15, HET = 13, KO = 14 from 5 animals/genotype; **b** Syn3/PSD95, *n* (sections analysed) WT = 9, HET = 9, KO = 9 from 3 animals/genotype **c** SV2/Homer, n (sections analysed) WT = 15 HET = 14 and KO = 14 from 5 animals/genotype. Statistical significance was determined by one-way ANOVA with Tukey’s multiple comparisons test, ns (not significant), **P < 0.01, ****P < 0.0001

In a complementary approach, we co-stained hippocampal sections with Syn1/Homer or Syn3/PSD95 presynaptic/postsynaptic marker pairs and estimated synaptic density in the hilus of the dentate gyrus (CA4) by co-occurrence of the pre- and postsynaptic markers. C9orf72 again significantly reduced the number of Syn1 and Syn3-positive excitatory synapses in both C9orf72-HET and C9orf72-KO mice compared to the C9orf72-WT controls (Fig. 5a, b). Furthermore, the levels of Syn1 and Syn3 were significantly reduced in the remaining synapses in C9orf72-HET and C9orf72-KO mice (Supplementary Fig. 8b, Online Resource). Hence in agreement with dissociated hippocampal neurons, loss of C9orf72 resulted in a decrease in synapsin-positive synapses and reduced synapsin levels. To verify if the effects observed were specific for synapsin we analysed SV2 stained hippocampal sections. Loss of C9orf72 led to a significant, gene dosage-dependent reduction in the SV2-positive area fraction and fluorescence intensity in the C9orf72-HET and C9orf72-KO mice compared to C9orf72-WT (Fig. 4c). Similarly, analysis of synaptic density in the hilus of the dentate gyrus (CA4) after co-staining with SV2 and Homer showed a significant reduction in SV2/Homer positive synapses upon loss of C9orf72 (Fig. 5c), and decreased levels of SV2 in remaining synapses (Supplementary Fig. 8b, Online Resource).

It is unlikely that neuron loss is responsible for the observed reductions in synapsin and SV2 staining because in agreement with previous reports [40, 87], we did not observe overt neuron loss in the hippocampus of the C9orf72-KO mice as analysed by determination of nuclei numbers in the dentate gyrus that innervates the mossy fibre area (Supplementary Fig. 8c, Online Resource). Hence, as was the case in vitro, loss of C9orf72 impairs hippocampal excitatory synapses in vivo.

### Synapsin-positive structures are reduced in the hippocampus of C9ALS/FTD patients

We and others have reported hippocampal involvement in C9ALS/FTD, including p62, DPR and TDP-43 inclusions [1, 14, 35, 51, 54, 75], and occurrence of hippocampal sclerosis [55, 60].

**Fig. 6:**
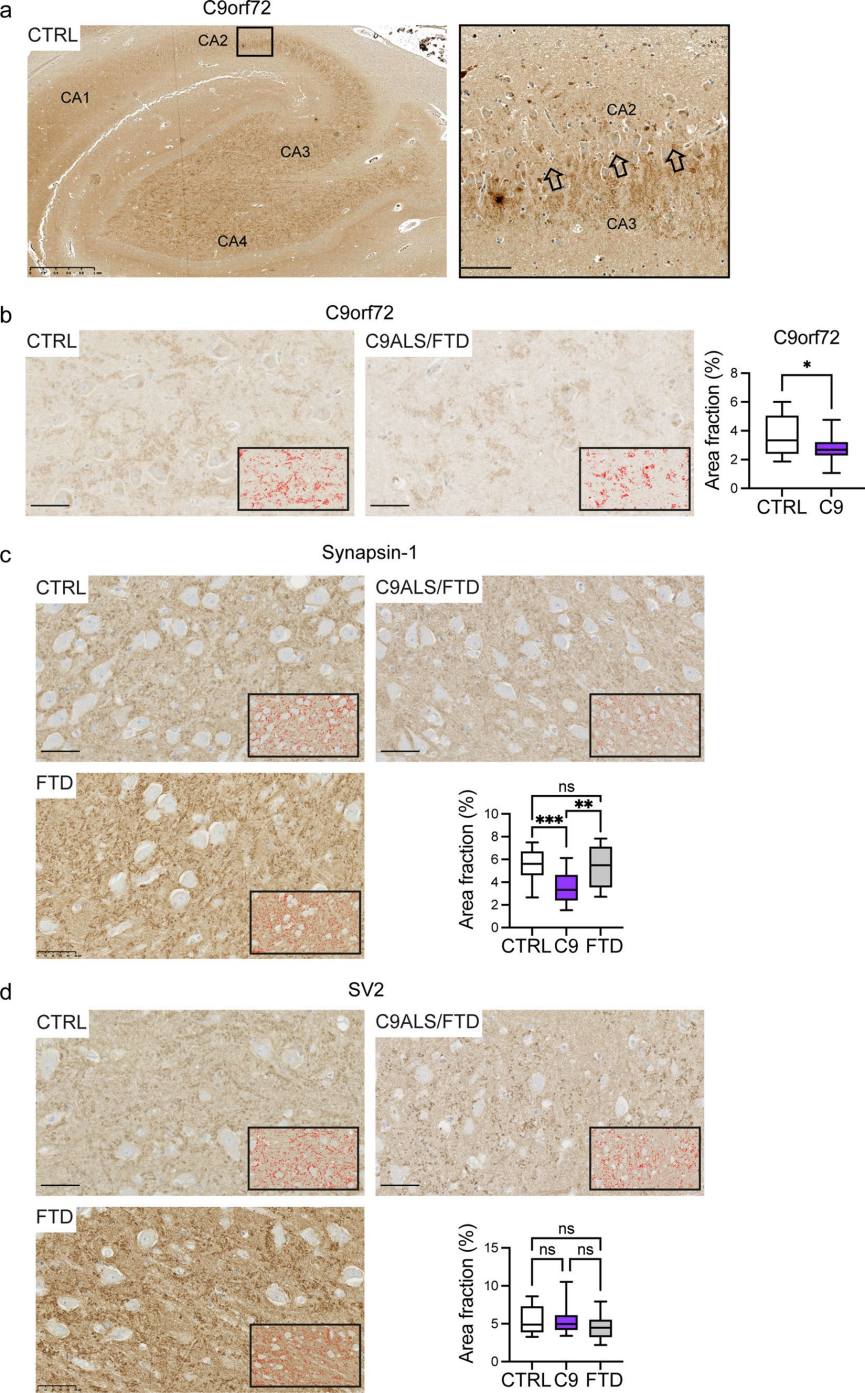
Levels of C9orf72 and synapsin-1 are reduced in C9ALS/FTD hippocampus. **a** Representative image of post-mortem immunohistochemistry staining for C9orf72 in the hippocampus of a neurologically healthy control (CTRL) brain with the CA1, CA2, CA3 and CA4 region indicated. Scale bar: 1 mm. Box denotes zoom area. Open arrows in zoom illustrate the interface between the CA3 and CA2 region. Scale bar 100 µm. **b** Representative images of C9orf72 staining in the hippocampus of a CTRL and a C9ALS/FTD case. Insert: red areas depict the automated colour threshold used for quantification of the staining. Scale bar 50 µm. The area fraction of positive staining was determined in two CA3 and two CA4 regions per case and is presented as box and whisker plots; n (CA3/Ca4 regions), CTRL = 12 from 3 cases, C9ALS/FTD = 20 from 5 cases. Statistical significance was determined by unpaired two-tailed *t* test, *P < 0.05. **c**–**d** Representative images of Syn1 (**c**) and SV2 (**d**) staining in the hippocampus of a CTRL, C9ALS/FTD and FTD case. Insert: red areas depict the automated colour threshold used for quantification of the staining. Scale bar 50 µm. The area fraction of positive staining was determined in two CA3 and two CA4 regions per case and is presented as box and whisker plots; *n* (CA3/Ca4 regions); Syn1 *n* (CA3/Ca4 regions) CTRL = 16 from 4 individuals, C9ALS/FTD = 20 from 5 cases, FTD = 12 from three cases; SV2 n (CA3/Ca4 regions) CTRL = 12 from 4 individuals, C9ALS/FTD = 20 of 5 cases, FTD = 12 from three cases. Statistical significance was determined by one-way ANOVA with Tukey’s multiple comparisons test, ns (not significant), **P < 0.01, ***P < 0.001

The data above indicate that loss of C9orf72 affects excitatory synapses. Therefore, we next examined whether the synaptic changes observed in vitro in dissociated hippocampal neuron cultures and in vivo in C9orf72-HET and C9orf72-KO mice were also observed in the brains of C9ALS/FTD patients with C9orf72 haploinsufficiency by immunohistochemistry of post-mortem hippocampal sections (Fig. 6). Demographic, clinical and pathological characteristics are provided in Table 2.

We first labelled sections using C9orf72 antibodies to confirm haploinsufficiency. We found that the CA3 and CA4 subregions of the hippocampus had stronger C9orf72 signal compared to other subregions of Ammon’s horn and the dentate gyrus. This differential staining was particularly obvious at the interface between the CA2 and CA3 subregion (Fig. 6a, open arrows in zoom). In CA3 and CA4 the staining appeared as granular labelling of the neuropil, the synaptically dense areas between neuronal cell bodies consisting of unmyelinated axons, dendrites and glial cell processes. Besides labelling of the neuropil, C9orf72 antibodies also stained granules resembling synaptic boutons around the pyramidal cells of this region (Fig. 6a). The area fraction stained positive for C9orf72 was significantly reduced by approximately 28% in CA3/CA4 sections of C9ALS/FTD patients compared to healthy controls, demonstrating C9orf72 haploinsufficiency (Fig. 6b).

Similar as observed for C9orf72, both Syn1 and SV2 antibodies produced granular labelling of the neuropil and granular staining around cell bodies, consistent with labelling of synapses and was most prominent in CA3 and CA4 (Fig. 6c, d). Compared to healthy controls Syn1 staining was significantly reduced by approximately 36% in C9ALS/FTD cases (Fig. 6c) whereas there was no significant difference in SV2 staining between groups (Fig. 6d). Hence, the main impairment appears to be loss of synapsin from synapses rather than a general loss in synapses. To ascertain if this effect was specific for C9ALS/FTD cases we further evaluated Syn1 and SV2 staining in non-C9orf72-related FTD cases. There was no significant difference in Syn1 or SV2 staining between these FTD cases and neurologically normal controls (Fig. 6c, d). Thus, disruption to synapses in the hippocampus is a specific feature of C9ALS/FTD.

### Loss of C9orf72 severely reduces the number of synaptic vesicles in excitatory synapses

**Fig. 7:**
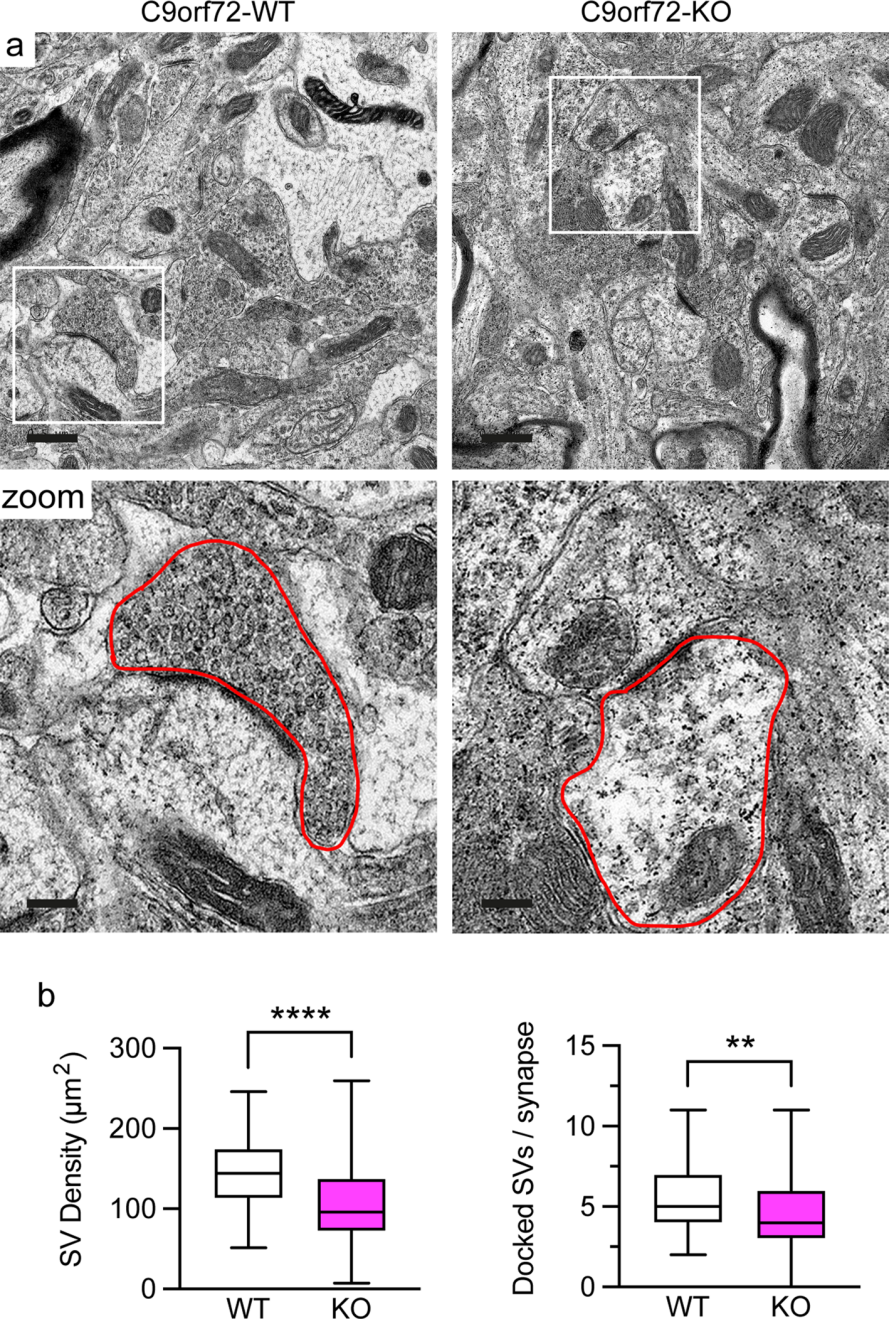
Loss of C9orf72 affects synaptic vesicle pools in the hippocampus in vivo*.* **a** Representative transmission electron micrographs of the CA3 region of the hippocampus from 12-week old C9orf72-WT or C9orf72-KO mice. Scale bar 0.5 μm. Box denotes zoomed area. An excitatory nerve terminal is outlined by the red line in the zoomed area. Scale bar 0.15 μm. **b** Quantification of the density of synaptic vesicles (SV density, μm^2^) and the number of docked synaptic vesicles per synapse (Docked SVs/synapse) in C9orf72-WT and -KO excitatory synapses. Data are is presented as box and whisker plots; *n* (synapses analysed) WT = 81, KO = 105 obtained from 4 C9orf72-WT and 5 C9orf72-KO animals. Statistical significance was determined by unpaired two-tailed *t* test, **P < 0.01, ****P < 0.0001

The data above established that C9orf72 interacts with synapsin and that loss of C9orf72 causes depletion of synapsin from synapses and synapse loss (Figs. 1, 2, 3, 4, 5, 6). Synapsins are known to regulate neurotransmission by controlling synaptic vesicle pools and ultrastructural synaptic abnormalities have been observed in synapsin deficiency [11, 70]. Therefore, loss of C9orf72 and subsequent depletion of synapsin from synapses may disrupt synaptic vesicle pools. To explore this possibility, we performed high resolution morphological studies using transmission electron microscopy (TEM) on hippocampi from 12-week-old C9orf72-WT and C9orf72-KO mice. As expected, in C9orf72-WT mice excitatory synapses in the hippocampal CA3 region were densely packed with synaptic vesicles that were distributed in distinguishable reserve and docked synaptic vesicle pools (Fig. 7a). In marked contrast, C9orf72-KO synapses appeared severely depleted of synaptic vesicles (Fig. 7a). Quantification confirmed that the overall density of synaptic vesicles was reduced by approximately 30% in synaptic terminals of C9orf72-KO mice compared to C9orf72-WT (Fig. 7b). Moreover, the number of morphologically docked synaptic vesicles, synaptic vesicles that are located immediately adjacent to the presynaptic membrane in the active zone, was reduced by approximately 20% in C9orf72-KO mice (Fig. 7b). Taken together these results show that loss of C9orf72 and concomitant reductions in synapsin in synapses severely affects the number of synaptic vesicles and therefore synaptic vesicle pools in excitatory synapses.

### C9orf72 haploinsufficiency affects excitatory neurotransmission and network function

Synapsin controls the storage and mobilisation of synaptic vesicles within the reserve pool and, furthermore, delivers synaptic vesicles to active zones and regulates neurotransmitter release and endocytosis [22, 26, 33, 70]. Given the depletion of synaptic vesicles from synapses in C9orf72-KO mice (Fig. 7) and loss of synapses and synapsin from remaining synapses in dissociated hippocampal neuron cultures treated with miRNA-C9 (Fig. 3), we hypothesised that C9orf72 haploinsufficiency may lead to decreased synaptic function and impaired neurotransmission.

**Fig. 8:**
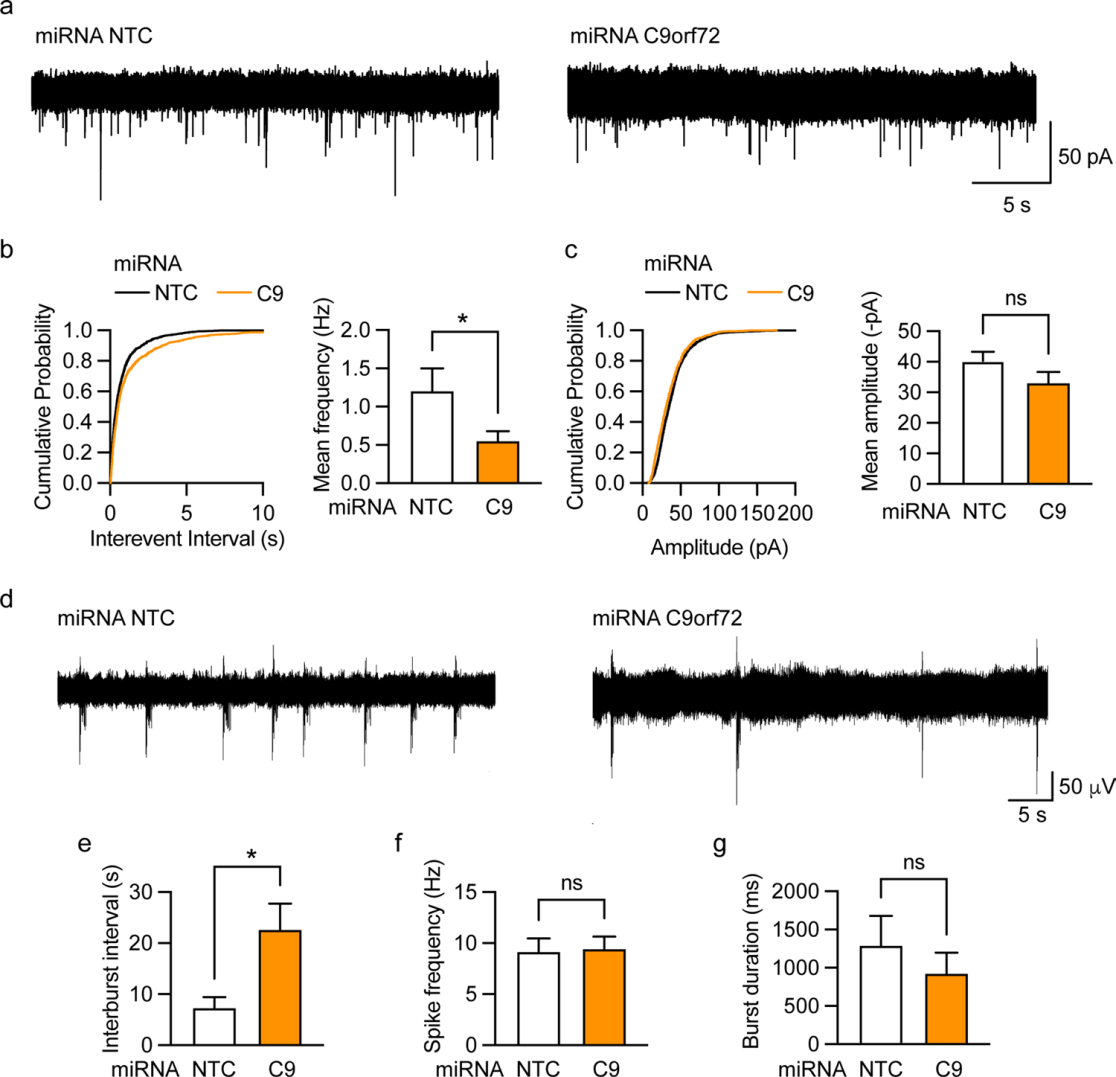
C9orf72 haploinsufficiency affects excitatory neurotransmission and network function. **a–c** Primary rat hippocampal neurons were transduced with non-targeting control miRNA (miRNA NTC) or C9orf72 miRNA (miRNA C9orf72) lentivirus at 5DIV and miniature excitatory postsynaptic current (mEPSC) traces were recorded from single neurons on 12–13DIV at a holding potential of –70 mV (–84 mV with liquid junction potential correction). **a** Representative current traces, **b** mEPSC interevent interval time and **c** mEPSC amplitude data are presented as cumulative probability and mean ± SEM of *n* = number of cells recorded n (cells) miRNA-NTC = 15, miRNA-C9 = 13 from three individual batches of neurons. Statistical significance was determined by unpaired two-tailed *t* test, ns (not significant), *P < 0.05. **d–g** Multi-electrode array (MEA) recordings to measure network activity were performed at 12–13DIV on hippocampal neuron cultures transduced with non-targeting control miRNA (miRNA-NTC) or C9orf72 miRNA (miRNA-C9) lentivirus at 5DIV. **d** Representative traces of a single array channel electrode recorded from miRNA NTC or miRNA C9-transduced neurons. Scale bars 5 s, 50 μV. Network activity characteristics were quantified by determining the **e** interburst interval of network bursts, **f** intra-network burst spiking frequency, **g** network burst length. Data are presented as mean ± SEM of *n* = number of MEA arrays, *n* (arrays) miRNA-NTC = 6, miRNA-C9 = 7 from 4 individual batches of neurons. Statistical significance was determined by unpaired two-tailed *t* test, ns (not significant), *P < 0.05

To test this hypothesis, we performed whole-cell voltage-clamp experiments on 12-13DIV hippocampal neurons in which C9orf72 expression was selectively reduced by lentiviral miRNA-C9 delivery (Supplementary Fig. 9a, b, Online Resource). To investigate functional synaptic properties, we measured miniature postsynaptic currents. Because our data indicated a specific effect of C9orf72 haploinsufficiency on excitatory but not on inhibitory synapses (Figs. 3, 6, Supplementary Fig. 7, Online Resource), we conducted our recordings in the presence of the voltage-gated sodium channel blocker tetrodotoxin (TTX) together with blockers of inhibitory transmission, namely picrotoxin (PTX, GABA_A_ receptor blocker) and strychnine (glycine receptor blocker), to isolate glutamatergic miniature excitatory postsynaptic currents (mEPSCs). mEPSC recordings in miRNA-C9 treated neurons revealed a substantial decrease in the frequency of mEPSC events by approximately 58% and a corresponding increase in the cumulative probability of the inter-mEPSC event interval compared to miRNA-NTC treated neurons (Fig. 8a, b). These results are consistent with a functional reduction in excitatory synaptic transmission in neurons haploinsuffient for C9orf72. Analysis of the individual mEPSC events showed that the postsynaptic current amplitude and decay kinetics were not significantly different in miRNA-NTC compared to miRNA-C9 treated neurons while the mEPSC rise time showed a modest increase when C9orf72 expression was reduced (Fig. 8c; Supplementary Fig. 9c, d, Online Resource). Because our experimental design favoured the isolation of AMPA receptor-mediated mEPSCs, these results indicate that the knockdown of C9orf72 did not impact upon the postsynaptic expression of glutamate-gated AMPA receptors. Thus, consistent with the reduction in excitatory synapses and loss of synapsin and synaptic vesicles in synapses (Figs. 3, 4, 5, 6, 7), the significant reduction of mEPSC frequency demonstrates that C9orf72 haploinsufficiency in hippocampal neurons affects the presynaptic compartment and functionally impairs excitatory neurotransmission.

In vitro cultured hippocampal neurons spontaneously develop functional network activity where action potentials (‘spikes’) become temporally organised into synchronized concentrated bursts. Synchronous bursts of network activity in these cultures rely on viable synaptic neurotransmission and network connectivity. Given the fact that we observed a substantially reduced frequency of synaptic mEPSC events in C9orf72-haploinsufficient neurons, we reasoned that this would impact upon neuron culture network activity.

To explore this, we cultured hippocampal neurons on multi-electrode arrays (MEA) that permit the detection of culture wide network activity and excitability. Network activity was measured on 12-13DIV hippocampal neurons in which C9orf72 expression was knocked down by treatment with lentiviral miRNA-C9 (Supplementary Fig. 9a, b, Online Resource) and assessed it according to a defined set of criteria (see Materials and Methods section).

We found that knockdown of C9orf72 increased the interval time between bursts (interburst interval) more than threefold, from 7.3 ± 2.1 s in miRNA-NTC to 22.6 ± 5.1 s in miRNA-C9-transduced neurons (Fig. 8d, e). C9orf72 haploinsufficiency did not affect the frequency of spikes within a burst or the actual duration of the burst (Fig. 8f, g). These data indicate that loss of C9orf72 reduced the basal network excitability of the cultures, which is in keeping with the reduction in excitatory synapses and loss of synapsin and synaptic vesicles in synapses we observed.

## Discussion

C9orf72 haploinsufficiency is hypothesised to contribute to the disease phenotype in C9ALS/FTD, however the underlying mechanisms are unclear. Despite recent reports showing that C9orf72 is present in synapses [21, 84], the function of C9orf72 in the CNS in general and in synapses in particular remains poorly understood. This study shows that C9orf72 plays a cell-autonomous role in the regulation of neurotransmission at excitatory synapses. We identified synapsin as a novel interactor of C9orf72 and explored the role of C9orf72 in presynaptic function using in vitro and in vivo C9orf72 loss of function models. We show that C9orf72 interacts with synapsin using Y2H, and endogenous co-immunoprecipitation and PLA assays. We present light and electron microscopic as well as electrophysiological evidence that C9orf72 is required for the maintenance of appropriate synaptic vesicle pools and neurotransmission. Furthermore, neuropathological analysis of post-mortem C9ALS/FTD patient brain samples replicated these findings, supporting the hypothesis that disruption of the interaction between C9orf72 and synapsin contributes to ALS/FTD pathobiology.

Synapsins are key proteins involved in the maintenance of the reserve pool of synaptic vesicles at the presynaptic terminal. Accordingly, perturbation or knockout of synapsins causes a dramatic reduction in synaptic vesicles in the reserve pool, particularly in excitatory synapses [28, 58, 62, 73] (Reviewed in [86]). Our data shows that C9orf72 loss of function causes reduced levels of synapsin at presynaptic sites in primary hippocampal neuron cultures as well as in the mossy fibre region of the hippocampus of C9orf72-KO mice and ALS/FTD patients (Figs. 3, 4, 5, 6). Moreover, direct observation of synaptic vesicle pools in C9orf72-KO mice by TEM revealed a striking reduction in the reserve pool in the same area (Fig. 7). Functionally we demonstrate that spontaneous synaptic activity in the form of mEPSCs is reduced in frequency in C9orf72 knockdown neurons (Fig. 8). This is consistent with reduced synaptic number (Fig. 3) and impaired availability of synaptic vesicles for fusion due to disturbed synaptic vesicle pools (Fig. 7). The remarkable resemblance between our TEM observations in C9orf72-KO mice, the mEPSC properties in C9orf72 knockdown neurons, and the reported phenotype of synapsin perturbation/KO [28, 58, 62, 73] (Reviewed in [86]), together with the direct interaction of C9orf72 with synapsin strongly implies that C9orf72 plays a role in the regulation of the synaptic vesicle reserve pool by its interaction with synapsin.

Despite clear evidence that synapsins maintain the reserve pool, the underlying mechanisms remain unclear. On one hand, synapsins have been proposed to anchor synaptic vesicles to each other by inter-vesicle dimerisation of membrane-associated synapsins. Alternatively, there is evidence that synapsins create a liquid phase that traps synaptic vesicles (Reviewed in [86]). We show that C9orf72 binds to the conserved C domain of synapsin (Fig. 2). The C domain is the primary site of synapsin/synapsin interaction, required for homo- and heterodimerisation [32]. How the interaction with C9orf72 affects synapsin-mediated regulation of the reserve pool remains to be determined but may thus involve effects on synapsin multimerisation. In this respect it is interesting to note that C9orf72 has been shown to regulate assembly and stability of mitochondrial electron transfer chain Complex I [79], suggesting it may play a similar role here.

In contrast to our observations in C9orf72-KO mice, triple-knockout of *SYN1*, *2*, and *3* did not affect the pool of docked vesicles at the active zone, suggesting that C9orf72 may regulate additional aspects of synaptic vesicle dynamics leading to reductions in the pool of docked synaptic vesicles. Rab3a is important for replenishment of docked synaptic vesicles in response to stimulation [44]. It is well established that C9orf72, in complex with SMCR8, regulates Rab GTPases [81], and Rab3a has been reported to interact with C9orf72 [21]. Thus, impaired Rab3a-mediated replenishment of docked synaptic vesicles may be the underlying cause of the reduction in docked synaptic vesicles in hippocampal synapses of C9orf72-KO mice we report here (Fig. 7). Indeed synapsin/Rab3a double KO mice show reduced docked synaptic vesicles as well as reserve pool depletion [13], similar to what we observed in C9orf72-KO mice (Fig. 7). Moreover, since synapsin has been identified as an effector of Rab3a and this interaction affects the function of both proteins [26, 27], our data indicate that C9orf72 sits at the crossroads of both pathways.

In agreement with our data revealing a role for C9orf72 in the regulation of synaptic vesicle pools, downregulation of SV2 and a reduced rate of synaptic vesicle cycling has been observed at the neuromuscular junction in a zebrafish C9orf72 loss of function model [10]. Reductions in SV2 and the size of readily releasable pool of vesicles were also reported in C9orf72-ALS patient-derived induced pluripotent stem cell (iPSC) cortical neurons [36, 57], but, while we observed reduced SV2 in hippocampal neurons in vitro after C9orf72 knockdown, and a C9orf72 gene dosage-dependent decrease in vivo in the hippocampus of C9orf72-HET and C9orf72-KO mice (Figs. 4, 5), we did not observe this in post-mortem hippocampus from C9ALS/FTD patients (Fig. 6). This may reflect differences between C9orf72 haploinsufficiency in humans and depletion of C9orf72 in mice or indicate that the pathophysiology of vesicle dynamics is an early feature of the disease. In line with our data showing a reduction in postsynapses (Fig. 3) reduced dendritic spine density has been reported in hippocampal neurons of C9orf72-KO mice which could be rescued by restoring ULK1-dependent autophagy [34]. A body of evidence has linked autophagy to synaptic transmission and the synaptic vesicle cycle [46] and we and others have shown that C9orf72 regulates autophagy [2, 3, 20, 34, 64, 74, 80, 81]. Hence it is possible that loss of C9orf72 elicits the synaptic deficits we observe here by disrupting autophagy. Alternatively, these are overlapping but independent functions of C9orf72 and problems in these pathways result in a ‘double hit’ to synapses. Along the same lines, disruption to the recently described function of C9orf72 in postsynapses [84] may conspire with the disruption to presynaptic function we reveal here.

How C9orf72 haploinsufficiency is involved in C9ALS/FTD is not yet clear. The data presented here show that C9orf72 haploinsufficiency per se can cause neuronal damage independent of gain-of-function mechanisms. Synaptic dysfunction and loss are a recurring theme in ALS/FTD, including in non-C9orf72 related familial and sporadic forms of the disease, and appear to precede neurodegeneration [31, 56, 57, 71]. Advanced stages of ALS/FTD, including in C9orf72 repeat expansion patients, are associated with synaptic loss, where the extent of loss correlates with the clinical severity of cognitive impairments [31]. Interestingly our data show that the hippocampal synaptic dysfunction we observed was specifically associated with C9ALS/FTD and C9orf72 haploinsufficiency, but not non-C9orf72-related FTD (Fig. 6). If this translates into a distinctive clinal phenotype in C9orf72 repeat expansion carriers is not yet clear, but it may be related to hippocampal sclerosis which was described in C9orf72 repeat expansion cases [7, 54, 55, 60]. Our data show that C9orf72 haploinsufficiency results in the loss of excitatory, but not inhibitory synapses (Fig. 3, Supplementary Fig. 7, Online Resource), and therefore implicate C9orf72 haploinsufficiency in neurons as a contributor to such synaptic loss via a cell-autonomous mechanism. We further show a reduced network bursting profile and therefore reduced network excitability in C9orf72 knockdown neuron cultures, consistent with diminished excitatory synaptic function and excitatory drive within our neuronal networks (Fig. 8). Our study therefore provides mechanistic evidence that C9orf72 haploinsufficiency contributes to a loss of neuronal function at the synaptic and network excitability level. In addition to the cell-autonomous synaptic function of C9orf72 reported here, a recent study reported synapse loss via non-cell-autonomous mechanisms in the motor cortex of C9orf72-KO mice [43]. Thus, C9ALS/FTD-associated C9orf72 haploinsufficiency likely contributes to synapse loss via both cell autonomous and non-cell autonomous mechanisms. In addition, synaptic loss has been also observed in a gain-of-function poly-GR (80-repeat) mouse model [12], and overexpression of GA DPRs has been shown to cause damage to synaptic vesicle release in cortical neurons [36], though the underlying mechanisms remain to be determined. Thus, in support of a ‘double hit’ model of C9ALS/FTD loss and gain-of function mechanisms may converge on synaptic dysfunction. In keeping with this, hippocampal neuron loss and exacerbated cognitive deficits were observed after inactivation of one or both endogenous C9orf72 alleles in mice expressing human transgenes carrying the repeat expansion [87].

It is worth nothing that polymorphisms in *UNC13A*, which encodes the presynaptic protein Munc13-1, that have been identified as risk factors for ALS/FTD [18, 59] cause loss of Munc13-1 function [9, 50]. Munc13-1 primes synaptic vesicles for exocytosis and is essential for fusion of synaptic vesicles [4]. Consequently, similar to our observations in C9orf72 knockdown neurons, loss of Munc13-1 reduces excitatory synaptic transmission [4]. Thus, a pattern emerges in which dysfunction of excitatory synaptic transmission increases ALS/FTD disease risk and suggests that targeting synapse loss and synaptic dysfunction may be a viable strategy in ALS/FTD.

Our study demonstrates that C9orf72 directly interacts with the synapsin family of presynaptic vesicle proteins, impacting upon their levels in synapses and disturbing their role in modulating vesicle trafficking and neuronal function. These data support previous observations that excitatory synaptic dysfunction increases disease risk in ALS/FTD, and demonstrate for the first time that C9orf72 haploinsufficiency significantly contributes to altered synaptic density, regulation and function in C9ALS/FTD.

Finally, our study reveals a potentially deleterious neurological effect of reducing C9orf72 protein levels, which should be taken into consideration when designing anti-sense oligonucleotide therapeutics targeting C9orf72.

## Supplementary Information

Below is the link to the electronic supplementary material.

Supplementary file1 (PDF 7158 kb)
