# Supplementary material for: An interaction between synapsin and C9orf72 regulates excitatory synapses and is impaired in ALS/FTD
Source: Acta Neuropathol. 2022 Jul 25;144(3):437–64. doi: 10.1007/s00401-022-02470-z (PMC9381633; doi:10.1007/s00401-022-02470-z)
Supplement: Supplementary file 1 — Supplementary file1 (PDF 7158 kb) [file 401_2022_2470_MOESM1_ESM.pdf]

Supplementary Figures

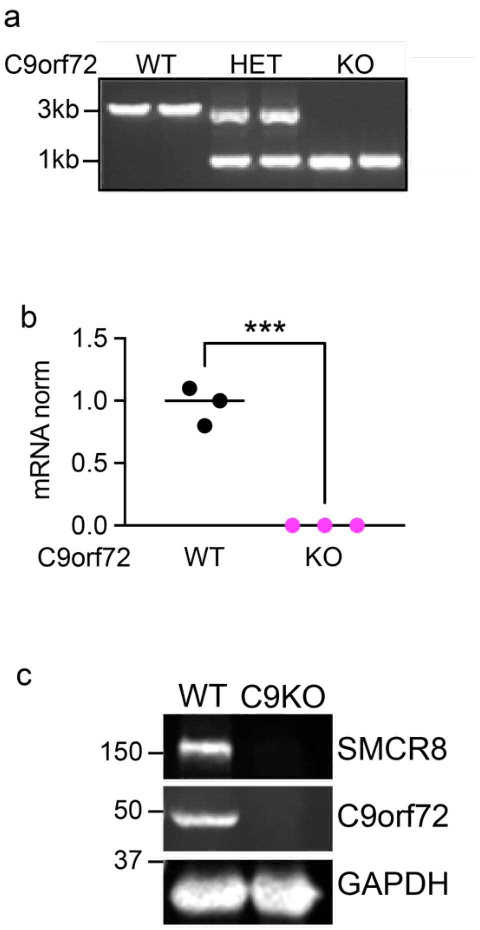

Supplementary Fig. 1 Characterization of the C9orf72 knockout mouse model.

a Qualitative PCR on RNA isolated from biopsies of wildtype (WT), heterozygous (HET) and homozygous (KO) mice confirming the excision of exons 3 and 4.

b Quantification of normalized C9orf72 mRNA levels in brain tissue of 8-week-old C9orf72-WT or C9orf72-KO mice measured by RT-qPCR confirmed the absence of C9orf72 mRNA in KO animals. Data are presented as mean ± SEM, statistical significance was determined by unpaired two-tailed t-test, *** $P < 0.001$.

c Western blot of brain lysates of 12-week-old C9orf72-WT or C9orf72-KO mice probed for SMCR8, C9orf72 and GAPDH as loading control confirmed the absence of C9orf72 protein in KO tissue. Note: SMCR8, a known interactor of C9orf72, was also reduced in KO animals.

a

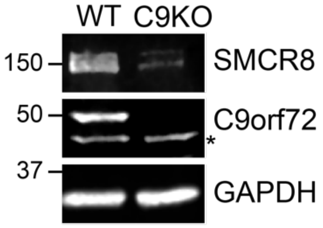

b

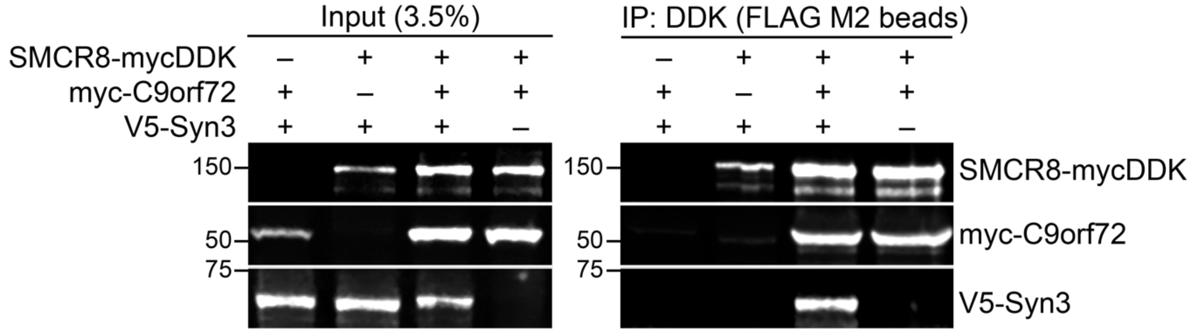

Supplementary Fig. 2 Syn3 does not interact with SMCR8.

a Immunoblot of lysates of HEK293 wildtype and HEK293 CRISPR/Cas9 C9orf72 knockout cells probed for SMCR8, C9orf72 and GAPDH as loading control confirmed the absence of C9orf72 protein in the knockout cells. Note: SMCR8, a known interactor of C9orf72, was also reduced in HEK293 CRISPR/Cas9 C9orf72 knockout cells. * indicates a nonspecific band.

b Cell lysates of HEK293 C9orf72 knockout cells co-transfected with either empty vector (EV) or SMCR8-mycDDK together with myc-C9orf72 and V5-Syn3a were subjected to immunoprecipitation using DDK (FLAG M2) magnetic beads. Immune pellets were probed for SMCR8-mycDDK, myc-C9orf72 and V5-Syn3a.

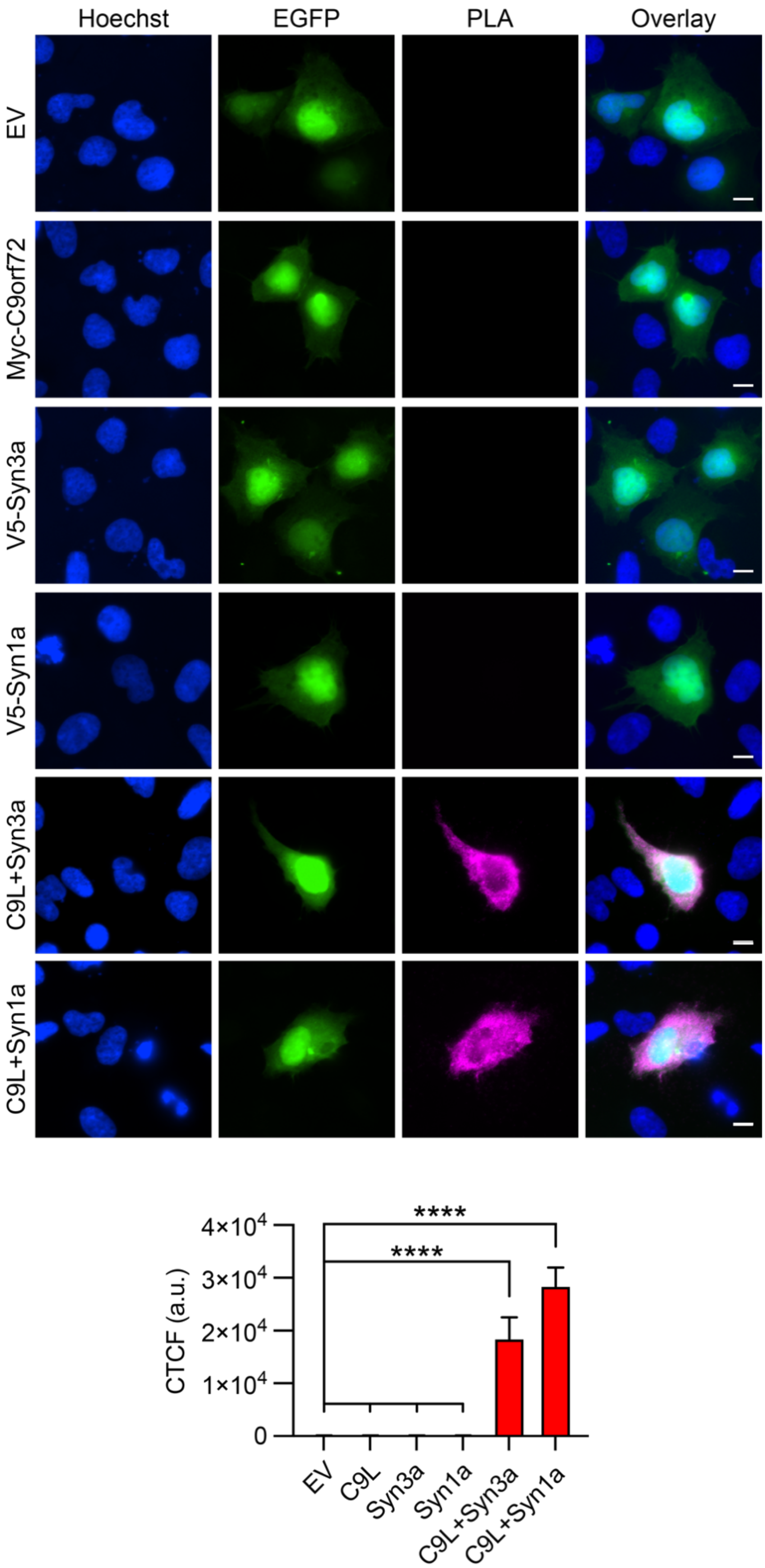

Supplementary Fig. 3 C9orf72 interacts with synapsin by PLA.

HEK293 cells were transfected with empty vector control (EV), Myc-C9orf72 (C9L), V5-Syn3a (Syn3a), V5-Syn1a (Syn1a), or co-transfected with either C9L+Syn3a or C9L+Syn1a. Transfections were laced with EGFP (green) to identify transfected cells. Samples were fixed, immunostained with antibodies against the Myc- or V5-tag or both antibodies together and processed for proximity ligation assay (PLA, magenta), nuclear staining with Hoechst (blue). Images are representative of the individual channels and their overlay. Scale bar: 10 µm. PLA intensity of transfected cells was analysed as Corrected Total Cellular Fluorescence (CTCF, arbitrary units (a.u.)). Data are presented as mean ± SEM, n (cells analysed) EV = 107, C9L = 92, Syn3a = 113, Syn1a = 79, C9L+Syn3a = 129, C9L+Syn1a = 109 from three or 4 replicate experiments. Statistical significance was determined by one-way ANOVA with Tukey's multiple comparisons test, **** $P < 0.0001$.

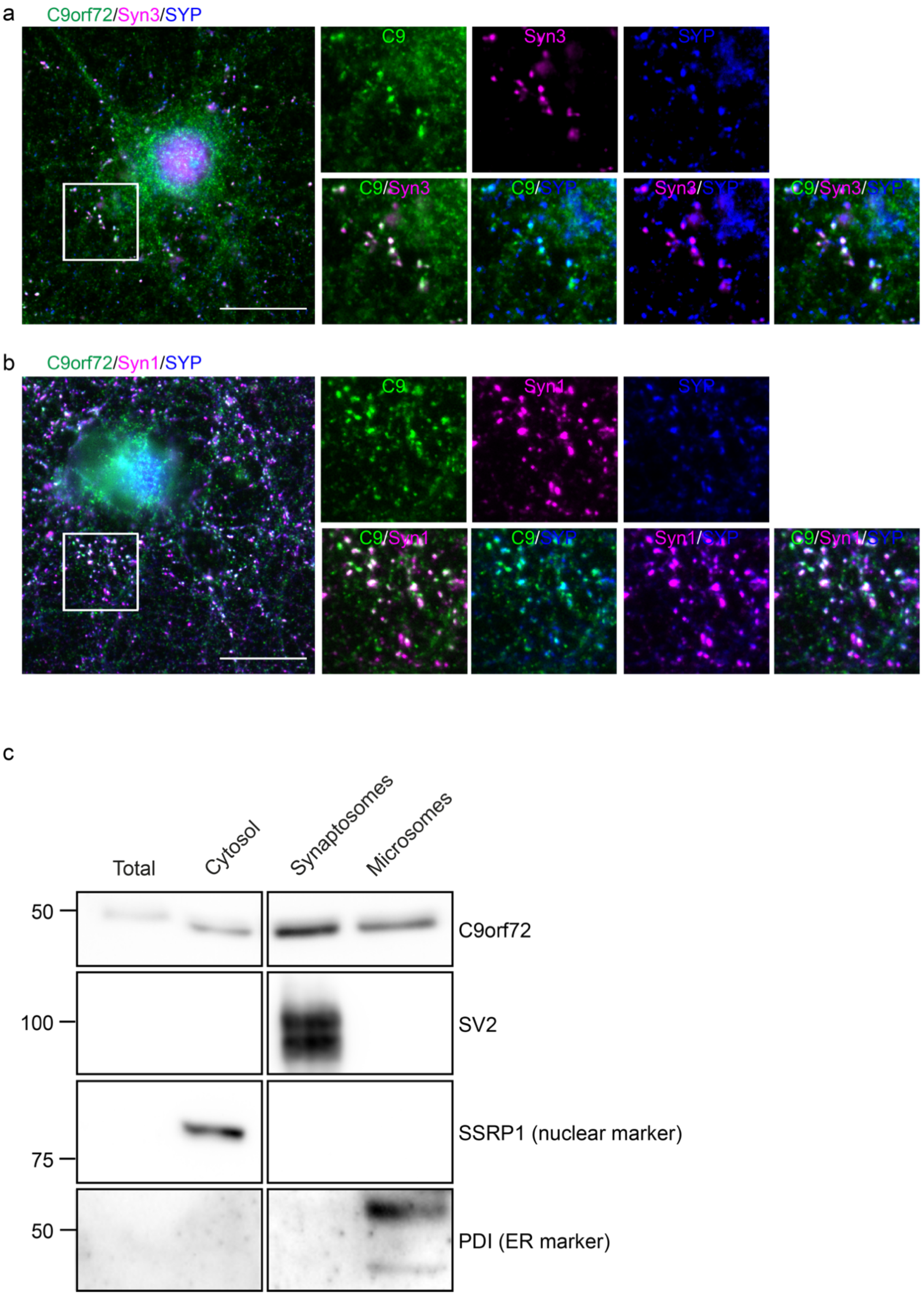

Supplementary Fig. 4 C9orf72 is enriched in synapses.

a 12DIV primary rat hippocampal neurons were immunostained with antibodies against C9orf72 (green), Syn3 (magenta) and synaptophysin (SYP, blue). Overlay images show partial co-localisation of all three proteins. Scale bar: 20 µm.

b 12DIV primary rat hippocampal neurons were immunostained with antibodies against C9orf72 (green), Syn1 (magenta) and synaptophysin (SYP, blue). Overlay images show partial co-localisation of all three proteins. Scale bar: 20 µm.

c Whole mouse brain lysates were fractionated into synaptosomes, microsomes and cytosol. To verify fractions, immunoblots were probed for endogenous C9orf72, the synaptic vesicle marker SV2, an antibody against the nuclear marker SSRP1 and the endoplasmic reticulum (ER) marker PDI. C9orf72 was enriched in the synaptosomal fraction.

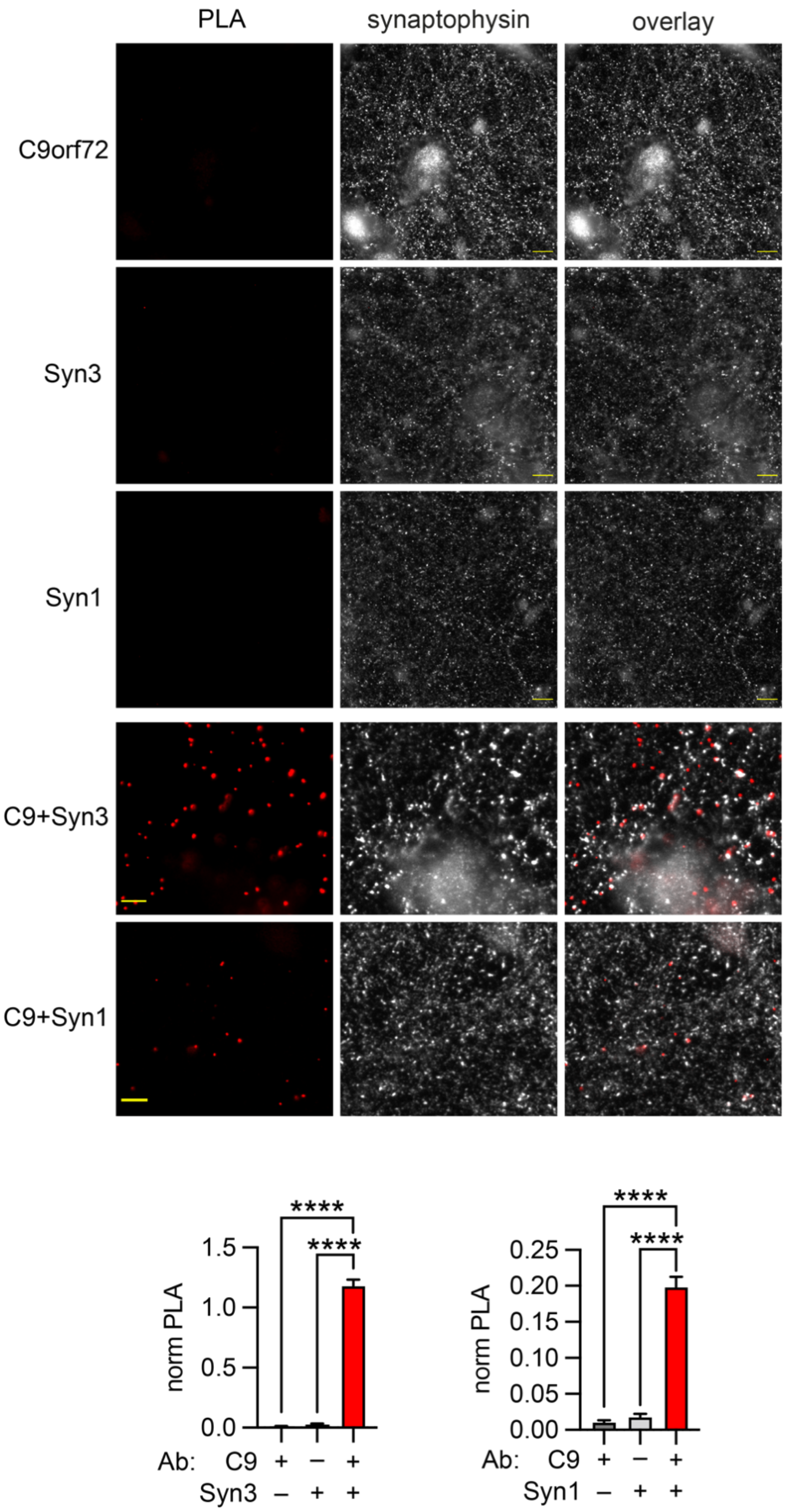

Supplementary Fig. 5 Endogenous C9orf72 interacts with endogenous synapsin by PLA. Single antibody PLA controls of 12DIV primary rat hippocampal neurons immunostained with antibodies against endogenous C9orf72, Syn3 or Syn1 or with pairs of antibodies against C9orf72 and Syn3 (C9+Syn3) or C9orf72 and Syn1 (C9+Syn1). All samples were co-stained

for endogenous synatophysin (white) and processed for proximity ligation assay (PLA, red). Scale bar: 5 µm. The number of PLA spots per image was normalized (norm PLA) to the mean grey intensity of the synaptophysin image. Data are presented as mean ± SEM; n (images analysed) C9 = 14, Syn1 = 14, Syn3 = 15, C9+Syn1 = 59, C9+Syn3 = 62 from two or three replicate experiments. Statistical significance was determined by one-way ANOVA with Tukey's multiple comparisons test, **** $P < 0.0001$.

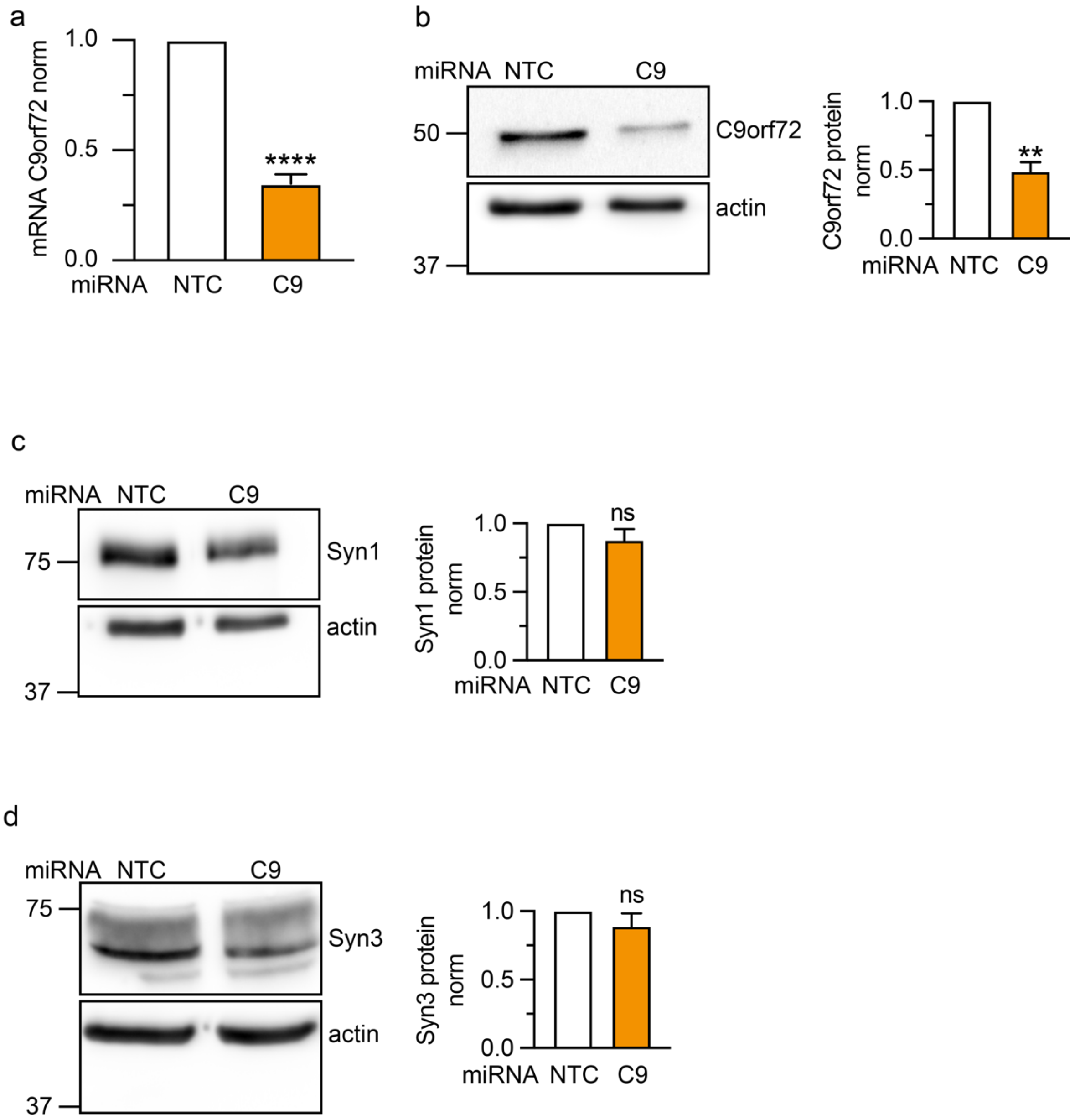

Supplementary Fig. 6 Lentiviral-mediated knockdown reduces mRNA and C9orf72 protein without affecting global Syn1 and Syn3 protein levels.

Primary rat hippocampal neurons were transduced with EmGFP non-targeting control miRNA (NTC) or C9orf72 miRNA (C9) lentivirus at 5DIV and total cell lysates harvested at 12DIV.

a Quantification of lentiviral-mediated knockdown of C9orf72 mRNA by RT-qPCR for n = 8 individual batches of neurons. Data are presented as mean ± SEM; statistical significance was determined by one sample t and Wilcoxon test, **** P < 0.0001.

b-d Western blot quantification of levels of C9orf72 (n = 7 batches), Syn1 (n = 5 batches), and Syn3 (n = 5 batches) normalized to actin in neuron lysates following lentiviral-mediated knockdown of C9orf72. Data are presented as mean ± SEM; statistical significance was determined by one sample t and Wilcoxon test, ns (not significant), ** P < 0.01.

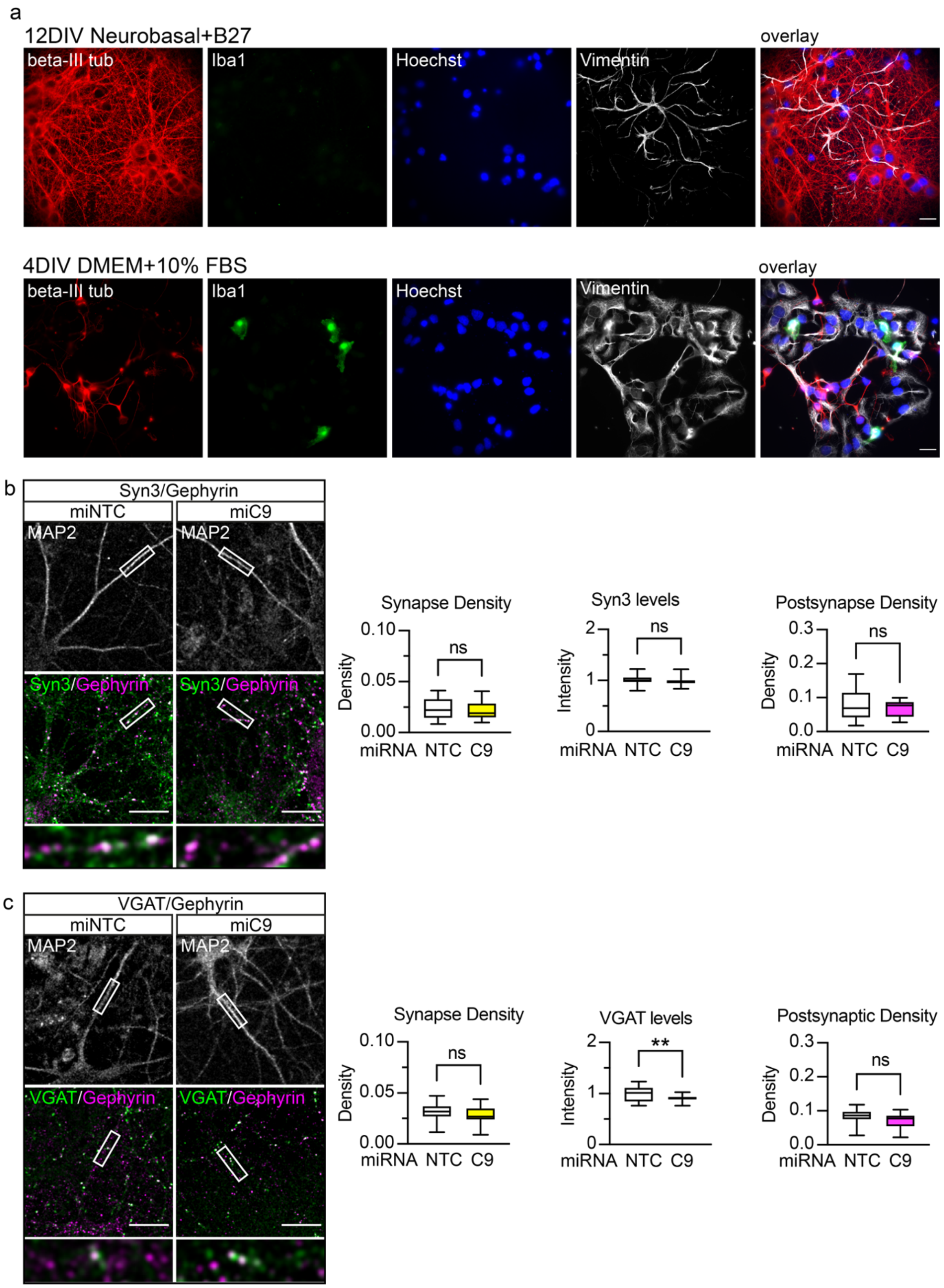

Supplementary Fig. 7 C9orf72 haploinsufficiency does not affect the number of inhibitory synapses.

a Representative images of 12DIV primary rat hippocampal cell cultures grown under standard neuron culture conditions as used in this study (Neurobasal medium supplemented with B27) immunostained for the neuronal marker beta-III tubulin, the microglia marker Iba1 and for Vimentin, a non-neuronal marker. Scale bar: 20 µm. Images are an example of a total of n = 16 images taken of two individual cultures.

Representative images of 4DIV primary rat hippocampal cell cultures grown under conditions supporting non-neuronal cell growth (DMEM supplemented with 10% FBS) demonstrate Iba1 antibody staining. Scale bar: 20 µm.

b Primary rat hippocampal neurons were transduced with EmGFP non-targeting control miRNA (miNTC) or C9orf72 miRNA (miC9) lentivirus at 5DIV. Confocal images of neurons immunostained at 12DIV for the dendritic marker MAP2 (white) and overlay images of presynaptic synapsin 3 (green) and postsynaptic gephyrin staining (magenta). Scale bar: 10 µm. Boxes indicate zoomed areas, note: co-localisation of pre- (green) and postsynaptic marker (magenta) appears white. Quantification of synapse density, intensity of presynaptic staining (Syn3 levels), and postsynapse density following image analysis using CellProfiler algorithms. Data are presented as box and whisker plots, n (images analysed) miRNA NTC = 30, miRNA C9 = 27 from three replicate experiments. Statistical significance was determined by unpaired two-tailed t-test, ns (not significant).

c Confocal images of neurons immunostained at 12DIV for the dendritic marker MAP2 (white) and overlay images of presynaptic VGAT (green) and postsynaptic gephyrin staining (magenta). Scale bar: 10 µm. Boxes indicate zoomed area, note: co-localisation of pre- (green) and postsynaptic marker (magenta) appears white. Quantification of synapse density, intensity of presynaptic staining (VGAT levels), and postsynapse density following image analysis using CellProfiler algorithms. Data are presented as box and whisker plots, n (images) miRNA NTC = 21, miRNA C9 = 22 from two replicates. Statistical significance was determined by unpaired two-tailed t-test, ns (not significant).

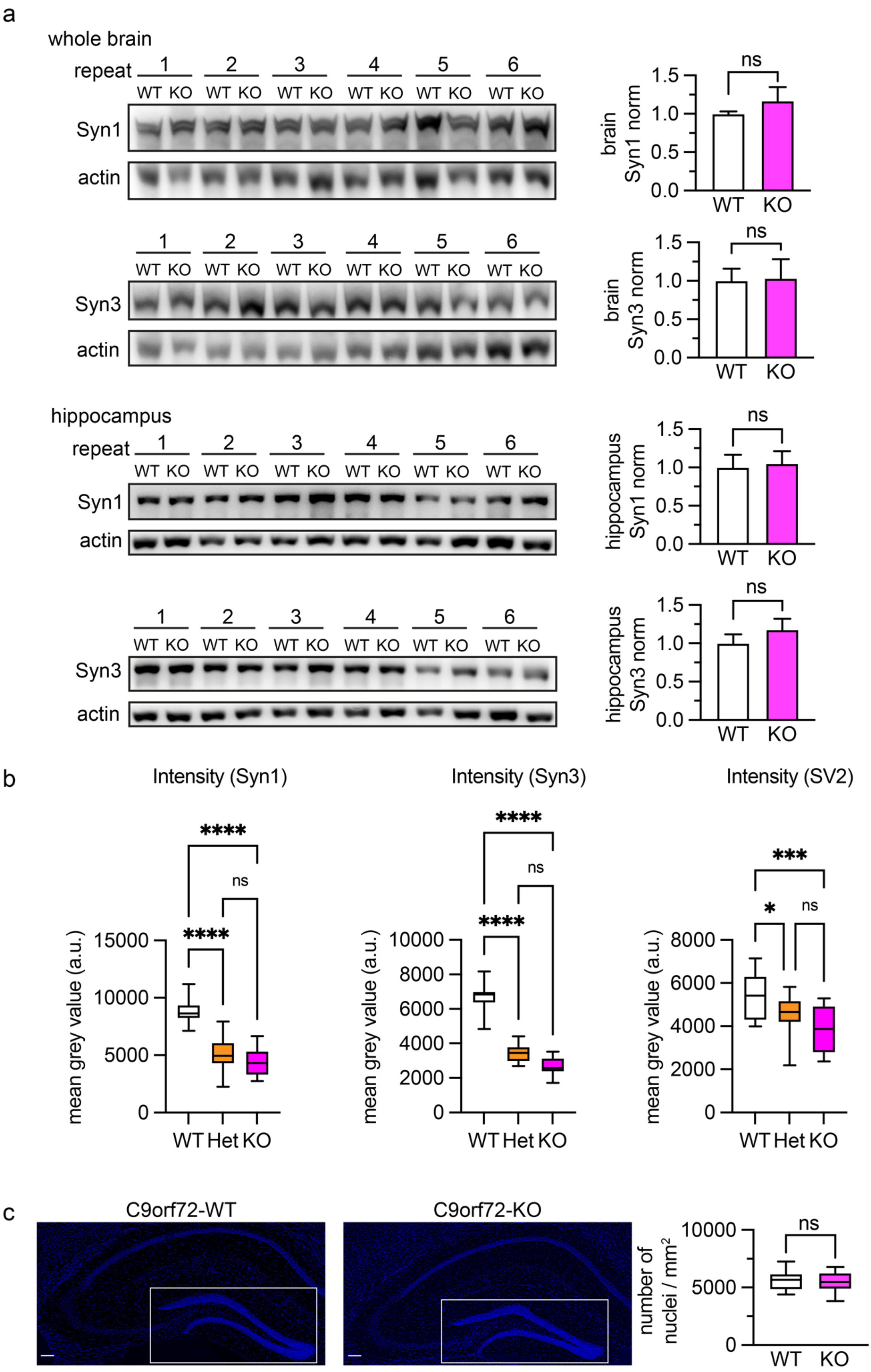

a
whole brain
repeat
1 2 3 4 5 6
WT KO
Syn1
actin
Syn3
brain Syn1 norm
brain Syn3 norm
ns
WT KO
hippocampus
hippocampus Syn1 norm
hippocampus Syn3 norm
b
Intensity (Syn1)
Intensity (Syn3)
Intensity (SV2)
mean grey value (a.u.)
WT Het KO
****
***
*
ns
c
C9orf72-WT
C9orf72-KO
number of nuclei / mm2

Supplementary Fig. 8 Loss of C9orf72 does not affect total synapsin levels and does not cause overt hippocampal degeneration.

a Immunoblot of whole brain or hippocampus lysates of 22-week-old C9orf72-WT or C9orf72-KO mice probed for synapsin-1 (Syn1), synapsin-3 (Syn3) and actin. Quantification of normalized levels of Syn1 and Syn3 in C9orf72-WT and -KO brain samples demonstrates that levels of synapsin were not significantly changed in C9orf72-KO animals. Data are presented as mean ± SEM of n = 6 animals. Statistical significance was determined by unpaired two-tailed t-test, ns (not significant).

b The mean fluorescence intensity level (Intensity) of Syn1, Syn3, and SV2 levels within synapses of detected by co-occurrence of Syn1/Homer**,** Syn3/PSD95 or SV2/Homer pre- and postsynaptic marker pairs, respectively, in the hilus of the dentate gyrus (CA4) region of the hippocampus of 12-week-old C9orf72-WT, C9orf72-HET and C9orf72-KO mice was determined. Data are presented as box and whisker plots; (**a**) Syn1, n (sections analysed) WT = 15, HET= 13, KO = 14 from 5 animals/genotype; (**b**) Syn3, n (sections analysed) WT = 9, HET = 9, KO = 9 from 3 animals/genotype (**c**) SV2, n (sections analysed) WT = 15 HET = 14 and KO = 14 from 5 animals/genotype. Statistical significance was determined by one-way ANOVA with Tukey's multiple comparisons test, ns (not significant), * $P < 0.05$, *** $P < 0.001$, **** $P < 0.0001$.

c Representative images of nuclear staining (blue) in sections of the hippocampus of C9orf72-WT or C9orf72-KO mice. Outlined box illustrates area of the dentate gyrus where the nuclei density (nuclei / mm^2) was analysed. Scale bar: 100 μm. Data are presented as mean ± SEM of n = 15 sections from 5 animals per genotype. Statistical significance was determined by unpaired two-tailed t-test, ns (not significant).

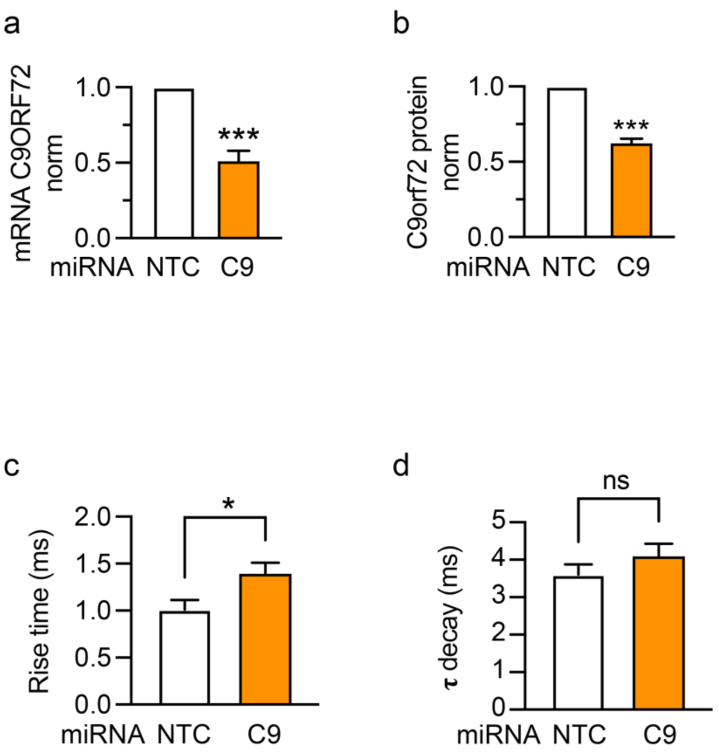

Supplementary Fig. 9 Lentiviral miRNA-mediated knockdown of C9orf72 and its effect on neurotransmission.

a Quantification of lentiviral-mediated knockdown of C9orf72 mRNA by RT-qPCR at 12DIV for n = 6 individual batches of transduced neurons used for patch-clamp and / or MEA recordings. Data are presented as mean ± SEM; statistical significance was determined by one sample t and Wilcoxon test, *** $P < 0.001$.

b Corresponding quantification of C9orf72 protein by Western blot. Data are presented as mean ± SEM of n = 5 of the batches; statistical significance was determined by one sample t and Wilcoxon test, *** $P < 0.001$.

c, d Analysis of kinetic parameters of miniature excitatory postsynaptic current (mEPSC) events. Rise time (10-90%, ms) and decay time (τ decay, ms) of mEPSCs recorded in miRNA NTC and miRNA C9 transduced neurons. Data are presented as mean ± SEM of n = number of cells recorded, n (cells) miRNA NTC = 15, miRNA C9 = 13 from 3 individual neuron batches. Statistical significance was determined by unpaired two-tailed t-test, * $P < 0.05$, ns (not significant) $P > 0.05$.

Supplementary Table 1 C9orf72 knockdown does not affect inhibitory presynaptic area

Staining	miRNA-NTC			miRNA-C9orf72			P value
	Area	SD	n	Area	SD	n	
Syn3/Gephyrin	14.45	2.38	30	13.7	2.49	27	0.2831 (ns)
VGAT/Gephyrin	18.66	2.67	21	17.9	2.40	22	0.3026 (ns)

The area of inhibitory presynapses was quantified in 12 DIV primary rat hippocampal neurons that had been transduced with EmGFP non-targeting control miRNA (NTC) or C9orf72 miRNA. Presynapses were labelled by immunostaining against Syn3 or VGAT and co-stained with the inhibitory postsynaptic marker Gephyrin to allow identification of inhibitory synapses. Data are presented as the mean and standard deviation (SD) of n images obtained from three (Syn3/Gephyrin) or two (VGAT/Gephyrin) independent experiments. Statistical significance was determined by unpaired two-tailed t-test; ns, not significant

Supplementary Materials and Methods

HEK 293 CRISPR/Cas9 C9orf72 knockout cell line

C9orf72-targeted hSpCas9n D10A nickase plasmids were generated according to the Zhang lab protocol [3]. Briefly, *C9orf72*-targeted DNA oligo pairs were annealed and cloned into pSpCas9n(BB)-2A-Puro (PX462) V2.0 (A gift from Prof Feng Zhang (MIT McGovern Institute, USA) via Addgene (Addgene plasmid #62987; http://n2t.net/addgene:62987; RRID:Addgene_62987) [3] via BbsI sites to produce pSpCas9n(BB)-2A-Puro nickase plasmids targeting the C9orf72 sense and C9orf72 antisense strand. C9orf72 targeting oligos were as follows: Sense: 5'-taacacatataatccggaa-3', Antisense: 5'-acacactctatgaagtggg- 3'. Each oligo was immediately followed by an NGG PAM site on the target sequence, allowing for the production of single strand nicks on opposing DNA strands. All plasmids were confirmed by sequencing.

To generate HEK293 C9orf72 knockout clones, cells were co-transfected with the sense and antisense C9orf72-targeting pSpCas9n(BB)-2A-Puro nickase plasmids in a 1:1 ratio using Lipofectamine 2000 reagent according to the manufacturer's instructions (Invitrogen, Thermo Fisher Scientific). 24 h post transfection cells were re-plated at 50% confluency and cultured in presence of 3 µg / ml puromycin to select transfected cells. 72 h post selection cells were re-plated in serial and limiting dilutions across 96 well plates to select for single clones. Clones were screened by immunoblot analysis for levels of C9orf72.

To confirm successful gene editing of the CRISPR HEK293 C9orf72 knockout line, we performed DNA sequencing of C9orf72 exon 2. For this genomic DNA was extracted using QuickExtract™ DNA extraction solution (Lucigen, Cambridge Bioscience, Cambridge, UK) and exon 2 was amplified by PCR using Phusion High Fidelity enzyme (NEB) with the following primers: C9orf72 exon 2 forward: 5'-AAATCATTTGGGGTTTTGATGG-3' C9orf72 exon 2 reverse: 5'-TGTAAAACGACGGCCAGTACCA-3'. The PCR product was subsequently cloned into the pCR-Blunt II-TOPO vector. HEK293 cells are hypotriploid with three alleles in the parental HEK293 line [1, 2]. Our CRISPR HEK293 C9orf72 knockout line was found to have a deletion in all three alleles of C9orf72; of either 4, 10 or 35 base pairs (bp), each of which resulted in a premature stop codon.

Absence of C9orf72 protein was confirmed on immunoblot of total cell lysates (Supplementary Fig. 1). Cells were lysed in BRB80 buffer (80 mM K-PIPES, 150 mM NaCl, 1 mM MgCl2, 1 mM EDTA, 1 % (w/v) NP-40, 1 x Halt™ Protease Inhibitor Cocktail) for 15 min at 4°C and lysates were cleared by centrifugation at 15,000 x g for 20 min.

Supplementary References

1. Bylund L, Kytölä S, Lui WO, Larsson C, Weber G (2004) Analysis of the cytogenetic stability of the human embryonal kidney cell line 293 by cytogenetic and STR profiling approaches. Cytogenet Genome Res 106:28-32. https://doi.org/10.1159/000078556

2. Lin YC, Boone M, Meuris L, Lemmens I, Van Roy N, Soete A et al (2014) Genome dynamics of the human embryonic kidney 293 lineage in response to cell biology manipulations. Nat Commun 5:4767. https://doi.org/10.1038/ncomms5767

3. Ran FA, Hsu PD, Wright J, Agarwala V, Scott DA, Zhang F (2013) Genome engineering using the CRISPR-Cas9 system. Nat Protoc 8:2281-2308. https://doi.org/10.1038/nprot.2013.143
